# Supplementary material for: Which outcomes are important to patients and families who have experienced paediatric acute respiratory illness? Findings from a mixed methods sequential exploratory study
Source: BMJ Open. 2017 Dec 14;7(12):e018199. doi: 10.1136/bmjopen-2017-018199 (PMC5736044; doi:10.1136/bmjopen-2017-018199)
Supplement: Supplementary file 1 [file bmjopen-2017-018199supp001.pdf]

Supplementary File 1

1. **We are interested in learning which things you would be most concerned about if your child was sick with an illness that affected their breathing. By moving each item from the left side of the screen to the right side of the screen, please choose the top five things that would be most concerning to you. In your list, put the thing that would be most concerning to you at the top, followed by the next most concerning thing, and so on.** *(randomize order)*

__ If your child needed to see a doctor
__ How long your child needed to stay in the emergency department or hospital
__ If your child needed to return to the doctor or hospital after being sent home
__ Your child's test results that tell the doctor how he or she is doing
__ Your child's reaction to his or her medicine (for example, if it caused side effects)
__ Your child's symptoms (for example, coughing, fever, sore throat)
__ The costs of your child's illness (for example, for medicine or child care)
__ How well you and your child could keep up the day-to-day activities of your normal life
__ That your child could develop minor complications from their illness, such as a cough or rash
__ That your child could have major complications from their illness, such as a long-term disability

---

We would like to further explore the things that matter to you, as a parent, when your child is sick with an illness that affects their breathing. For the questions below, use the sliding scale to show how concerned you would be in each of the situations described.

Not at all concerned--*Very concerned* __ *Can't answer*
(this would not be an important concern for you if your child was sick) (this would be a very important concern for you if your child was sick)

<u>**Getting Medical Help**</u>

Sometimes when children are sick and their breathing is affected, they need help from a doctor. How concerned would you be about the following: *(Image 1)*

1. That you had to take your child to your family doctor or pediatrician's office.
2. That you had to take your child to the emergency department.
3. How long you had to stay at the emergency department.
4. That your child had to stay in the hospital (overnight or longer).
5. How long your child had to stay in the hospital.
6. That your child needed a scheduled follow-up visit, either with your family doctor/pediatrician or at the hospital.
7. That you had to go back see a doctor after the first time because he or she was not getting better. This could be at a doctor's office, a clinic, or the hospital.

<u>**Tests and Treatment**</u>

Sometimes children need tests when they are sick. How important are the following things to you: *(sliding scale: Not at all important – Very important) (Image 4)*

8. Assessment of things like how fast your child was breathing, how fast his or her heart was beating, or whether he or she had a fever.
9. Results from x-rays, blood tests, or other tests, showing how well your child's lungs were working or what might be causing their illness.
10. The doctor's assessment of how your child was doing.

Sometimes when children are sick, they need to take medicine or be treated in other ways. How concerned would you be if: *(Image 5)*

11. Your child needed to be given extra oxygen through a mask or through his or her nose.
12. Your child needed to take medicine to feel better.
13. Your child had a minor but common side effect from the treatment or medicine. Some common side effects are vomiting, rash, and diarrhea.
14. Your child had a major side effect from the treatment or medicine that was bad enough to make him or her stop taking it. Some such reactions are your child becoming very agitated or irritable, or having a serious allergic reaction.

Other Concerns

Sometimes when children are sick, they are not themselves. How concerned would you be about the following things if your child was sick? *(Image 6)*

15. How bad your child's symptoms were. Symptoms are signs of your child's sickness. Common symptoms are things like coughing, wheezing, fever, sore throat, headache, and struggling to breathe.
16. How long it took for your child to feel better.
17. If your child got sick again with the same illness, after feeling better.

Sometimes when children are sick, parents or caregivers need to pay for extra things. How concerned would you be about: *(Image 7)*

18. How much your child's treatment cost, including costs for things related to treating their illness like medications, supplies, or doctor or nurse visits.
19. Other costs that came up while your child was sick, for example, child care, parking, or lost income for missing work.

Sometimes when children are sick, parents or caregivers can't follow their regular schedule or do regular activities. How concerned would you be about: *(Image 8)*

20. Your child not eating or drinking well because they felt sick.
21. You or your child not getting as much sleep as you normally do.
22. Your child being away from school or daycare.
23. The illness affecting the activities your child, you, or your family would normally do on a day-to-day basis.
24. Having to take time off of work.
25. Arranging child care either for your child who was sick, or arranging child care for your other children so that you could look after your sick child or take him or her to appointments.

It can sometimes be scary when our children are sick. *(Image 9)*

26. How concerned would you be if your child had mild complications such as a cough or a rash?
27. How concerned would you be if your child had severe complications? This could mean something like your child being connected to a ventilator to help his or her breathing.

---

28. **Please tell us any other things that are important to you when your child is sick with an illness that affects their breathing that have not been included in this list.**

Please tell us a little bit more about your family.

29. **What is your gender?**
    __ Male
    __ Female
    (__ NR)

30. **What is your relationship to the child(ren) in your family? Check all that apply.**
    __ Parent
    __ Step-parent
    __ Grandparent
    __ Other, specify: _________
    (__ NR)

31. **How many children live in your home?** ___ (__ NR)

32. **What illness or illnesses that affect breathing has your child or children ever had? Please check all that apply.**
    __ Bronchiolitis
    __ Croup
    __ Strep throat (pharyngitis) or tonsillitis
    __ Sinus infection (sinusitis)
    __ Wheezing
    __ Flu (influenza)
    __ Pneumonia
    __ Acute asthma
    __ Other, specify: _____________
    __ I don't remember the name of the illness
    (__ NR)

    33a. If you checked more than one illness above, which one did your child have **most recently**? *(drop down menu – same list as above)* (__ NR)

Supplementary File 1

*****The following three questions will appear to correspond to conditions above*****

33b**. How old was your child when they had this illness?** *(drop down menu - <1/1/…/10/11 months/1/2/…18 years/can't remember)* (__ NR)

33b. When did this illness occur? MM/YYYY (__ NR)

33c. **Was your child admitted to the hospital, to stay overnight or longer?**

__ Yes
__ No
(__ NR)

33d. **Does your child have any health concerns?**

__ Yes, details: _________________
__ No
(__ NR)

33. **What year were you born in?** 19____ (__ NR)

34. **What is the highest level of schooling you have completed?**

__ Grades 1-9
__ Grades 10-11/Some high school
__ High school graduate
__ Some college/university
__ College/university graduate
__ Post-graduate education or degree
(__ NR)

35. **What is your marital status?**

__ Never married (single)
__ Married/Common-law
__ Separated, divorced, or widowed
__ Other, specify: _________
(__ NR)

36. **What is your household income (US dollars) per year?** (http://www.xe.com/currencyconverter/)

__ Less than $30,000 per year
__ $30,000-49,999
__ $50,000-69,999
__ $70,000-89,999
__ Over $90,000
(__ NR)

37. **What was your place of birth (town/city, country)?** _________ ________ (__ NR)

38. **Where do you live now (town/city, country)?** _______ ________ (__ NR)

39. **How did you hear about this survey?**

__ Facebook
__ Twitter
__ Internet search (for example, Google)
__ Consumer or patient group (for example, Cochrane Consumer Network)
__ From a friend
__ From a health care professional (for example, a doctor or nurse)
__ Research network (for example, TREKK [Translating Emergency Knowledge for Kids])
__ Other, specify: ____________________
(__ NR)

Thank you for taking the time to fill out this survey!

If you would like to be entered into a draw for an iPad mini, a Kobo Touch, or a Nike+ FuelBand, click here to provide us with your email address. Your email address will not be linked to the responses you have provided.
