# Supplementary material for: Which outcomes are important to patients and families who have experienced paediatric acute respiratory illness? Findings from a mixed methods sequential exploratory study
Source: BMJ Open. 2017 Dec 14;7(12):e018199. doi: 10.1136/bmjopen-2017-018199 (PMC5736044; doi:10.1136/bmjopen-2017-018199)
Supplement: Supplementary file 2 [file bmjopen-2017-018199supp002.pdf]

Supplementary File 2

OUTCH Discussion Guide

Important Considerations

1. Is there agreement on the outcomes chosen, both among parents involved in the discussion, and between the discussion and the survey?
2. Are there areas (i.e., in current research) that have been overlooked?
3. What is the rationale for outcome selection?
4. What are considered to be clinically important or meaningful differences in the outcomes selected?

Week 1: February 1 – 5, 2016

Theme: **Getting Medical Help**

Sometimes when children are sick and their breathing is affected, they need help from a doctor.

1. Tell us about a time you needed to bring your child to a doctor because their breathing was affected.
    a. Did you have to go to see a doctor? Go to the emergency department?
    b. How did you feel?
    c. Did, or would, one have more impact on you than the other?
2. Tell us about what you find most concerning about getting medical help for your child when they are sick and their breathing is affected.
    a. Possible considerations (as prompts):
        i. how long you had to stay at the emergency department;
        ii. how long you had to stay in the hospital;
        iii. if you had to go for a scheduled follow-up visit to your family doctor or pediatrician;
        iv. if you had to go back to see a doctor after the first time because your child wasn't getting better.

- Thanks for checking out the OUTCH Study! We want to make sure that your child's health care is based on what's most important to you when their breathing is affected. This week, tell us what matters when your child needs to see the doctor. [attach graphic]

- Hey Parents! Have you had to take your child to the doctor or emergency department because they were having a hard time breathing? What was important to you?

- Excited to get this discussion going about what is important to you when your child is having a hard time breathing! Check out our page and share your experiences in the comments section.

Week 2: February 8 – 12, 2016

Theme: **Medical Tests**

Sometimes children need tests and treatment when they are sick.

Supplementary File 2

1. Tell us about a time your child needed tests because they were sick and their breathing was affected.
    a. What kind of tests did they need?
    b. At the time, how much did the results of things like x-rays or blood tests matter to you?
    c. How much did the doctor's assessment of your child's illness matter to you?
    d. Did, and why did, one type of assessment matter more to you than the other?

- Welcome to another week of the OUTCH Study! Help us understand your story. Tell us in the comments below about what matters to you when your child needs medical tests because they are having a hard time breathing. [attach graphic]

- If your child has needed tests like x-rays or blood tests when they're sick, what has mattered to you? Tell us in the comments below!

- Get to Know OUTCH!
We're asking you to share your stories with us, so we thought we'd tell you a little more about our team!

What does OUTCH stand for?
OUTCH stands for Outcomes in Child Health.

Who are we?
We're a group of researchers from the Department of Pediatrics at the University of Alberta, and we're the ones behind the scenes at the OUTCH Study.

What is the OUTCH Study all about?
We're interested in learning about outcomes that matter to you when your children's breathing is affected. This could mean that they have, or had, an illness like bronchiolitis, croup, strep throat or tonsillitis, a sinus infection, wheezing, the flu, pneumonia, or acute asthma. We want to talk to parents like you to make sure that our research asks the right questions, so we can improve healthcare for children and their families.

How can you help?
Tell us what matters to you in the comments section of our Facebook posts – we'd love to hear from you!

<u>Week 3: February 16 – 19, 2016 (*Family Day on February 15)</u>

Theme: **Medical Treatment**

Sometimes when children are sick, they need to take medicine or be treated in other ways.

1. Tell us about a time when your child was sick and they needed treatment because their breathing was affected.
    a. What kind of treatment did they need?
        i. Were they given medicine? Oxygen?
2. How concerned were you about your child needing treatment?
    a. What was most concerning about your child needing treatment because their breathing was affected?
3. If your child has had more than one kind of treatment, have you found certain treatments more concerning than others?
4. How concerned would you be concerned about a mild side effect from the treatment or medicine, like vomiting, a rash, or diarrhea?
5. How concerned would you be about a major side effect from the treatment or medicine (like being very agitated or having a serious allergic reaction), that was bad enough that your child had to stop taking it?

- Welcome back parents! Here in Alberta we're just coming off of our Family Day long weekend, but we hope you had a relaxing weekend wherever you are! This week, we're talking about medical treatment for your kids. What was your experience when your child was sick and needed treatment to help their breathing? What went well? What could have been better? What worried you? What was reassuring? Please tell us in the comments below!

- Medicine can help our kids a lot, but sometimes it comes along with annoying, or even scary, side effects. Has your child gone through this? Did it get bad enough to stop treatment, or were they okay to wait it out? Any lasting effects? A combination? Share your experience and how you felt in the comments below.

- What goes through your mind when your child is prescribed a treatment? Relief? Possible long-term effects? That it's the right one? A combination? Tell us your thoughts in the comments below!

<u>Week 4: February 22 – 26, 2016</u>

Theme: **Course of Illness**

Sometimes when children are sick, they are not themselves.

1. Tell us about symptoms you find the most concerning when your child is sick and their breathing is affected. *(Common symptoms are things like coughing, wheezing, fever, sore throat, headache, and struggling to breathe)* Why?
    a. What about things you're not too worried about? Why not?
2. How important to you is the amount of time that it takes your child to feel better?
    a. Is there a point in time when you're not concerned versus a point in time when you become concerned?

3. How concerned would you be if your child got sick again with the same illness, after feeling better?

- Sometimes when children are sick, they are not themselves. Respiratory infections can mean some nasty symptoms for kids, like coughs, fevers, and sore throats. What symptoms do you find most concerning when your child is sick? Tell us in the comments below!

- Parents, how do your dinosaurs get well soon?! Tell us what your kids are like when they're sick in the comments below!

- Respiratory infections can bring anyone down for a while. How do your kids cope? #BringBackFridayFunDay

Week 5: February 29 – March 4, 2016

Theme: **Financial Burden**

Sometimes when children are sick, parents or caregivers need to pay for extra things.

1. How concerned are you about the costs that come up when your child is sick?
   a. Costs related to treatment, like medications, supplies, or doctor or nurse visits?
   b. Other costs that came up while your child was sick, like child care, parking, or lost income for missing work?

- Happy Monday, Moms and Dads! This week, we're talking about costs that might come up when your kids are sick. Tell us what has come up for you in the comments below!

- There's a lot to think about when your child is sick. How much do you think about extra costs? Maybe that looks like costs for treatment, or maybe it's something like lost income for missing work. Share your thoughts in the comments below!

- Those darn hidden costs! Tell us what gets to you in the comments below!

Calvin and Hobbes, by Bill Watterson

Week 6: March 7 – 11, 2016

Theme: **Daily Activities and Routines**

Sometimes when children are sick, we can't follow a regular schedule or do regular activities.

1. Are there any regular activities that you'd be concerned about if your child couldn't do them when they're sick and their breathing is affected?
    a. How concerned would you be about your child:
        i. Not eating or drinking well?
        ii. Not getting as much sleep as they normally do?
        iii. Being away from school or daycare?
    b. How concerned would you be about:
        i. Having to take time off work?
        ii. Arranging child care for your sick child or other children?
2. Are there any other activities that you'd be concerned about disruptions to?

- Welcome to another week of the OUTCH Study! Thanks for checking us out. This week, we're wondering about how your regular routine is disrupted when your child is sick. What happens in your family that's out of the ordinary? Tell us below!

- Sound familiar? Are there activities that you and your little ones miss when they are sick?

Just a Little Sick, by Mercer Mayer

- Thanks for the great discussion this week! Sounds like sleep is at the top of a lot of parents' lists of routines that are disrupted when their child is sick - although "everything" would also probably cover it! What else happens in your family? Tell us in the comments below!

<u>Week 7: March 14 – 18, 2016</u>

Theme: **Complications**

It can sometimes be scary when our children have health complications because their breathing is affected

1. How concerned would you be if your child had mild complications like a cough or a rash?
    a. What was or would be most concerning about your child having mild complications?
2. How concerned would you be if your child had severe complications like needing to be connected to a ventilator to help them breathe?
    a. What was or would be most concerning about your child having severe complications when they were sick and their breathing was affected?

- Welcome back, Moms and Dads! Sometimes when children are sick, complications come up, which can be scary. Complications might be things like a cough or a rash, or could be more serious like needing to

be connected to a ventilator. Have your children gone through this? Please share your experience below.

- This week, we're talking about complications. Here's one mom's story:

He had all the signs of a cold coming on. With the rest of his siblings having been sick over the last few weeks, it was no surprise. He was super cranky all day and by supper time, he wasn't eating or drinking much. He'd had colds before but he wasn't acting well and looked tired. It all happened so fast - a drive to the hospital and 5 hours later, he was hooked up to a machine to help him breathe. The doctor said he had RSV and would likely need to stay a few days to be monitored and recover. I can't forget the helplessness I felt, watching him fighting to breathe, needing to be connected to the ventilator.

What about you? Please share your story in the comments below.

- Hey Parents, has your child had any complications when they've been sick with a respiratory infection? Tell us about it in the comments below.

Week 8: March 21 – 25, 2016

Theme: **Closing**

1. Please tell us any other things that are important to you when your child is sick with an illness that affects their breathing that we have not talked about yet.

- Happy Monday, everyone, and welcome to the last week of the OUTCH Study! Until next Sunday, tell us what matters to you when your child is sick with a respiratory infection. Is it their symptoms? Needing to see a healthcare provider? Disruptions to normal routines? Share what hits home for you in the comments below!

- Hey Parents, help us make sure we're not missing anything! What is most important to you when your child has a respiratory infection? Anything that's on your mind – we want to know!

- Thanks, everyone, for taking part in the OUTCH Study! Your input means a lot to us as we continue to try to make child health research and care better!
