# Supplementary material for: Which outcomes are important to patients and families who have experienced paediatric acute respiratory illness? Findings from a mixed methods sequential exploratory study
Source: BMJ Open. 2017 Dec 14;7(12):e018199. doi: 10.1136/bmjopen-2017-018199 (PMC5736044; doi:10.1136/bmjopen-2017-018199)
Supplement: Author's manuscript [file bmjopen-2017-018199.draft_revisions.pdf]

BMJ Open is committed to open peer review. As part of this commitment we make the peer review history of every article we publish publicly available.

When an article is published we post the peer reviewers' comments and the authors' responses online. We also post the versions of the paper that were used during peer review. These are the versions that the peer review comments apply to.

The versions of the paper that follow are the versions that were submitted during the peer review process. They are not the versions of record or the final published versions. They should not be cited or distributed as the published version of this manuscript.

BMJ Open is an open access journal and the full, final, typeset and author-corrected version of record of the manuscript is available on our site with no access controls, subscription charges or pay-per-view fees (http://bmjopen.bmj.com).

If you have any questions on BMJ Open's open peer review process please email editorial.bmjopen@bmj.com

BMJ Open

Which outcomes are important to patients and families who have experienced pediatric acute respiratory illness? Findings from a social media engagement strategy

Journal:	*BMJ Open*
Manuscript ID	bmjopen-2017-018199
Article Type:	Research
Date Submitted by the Author:	13-Jun-2017
Complete List of Authors:	Dyson, Michele; University of Alberta, Department of Pediatrics<br>Shave, Kassi; University of Alberta, Department of Pediatrics<br>Gates, Allison; University of Alberta, Department of Pediatrics<br>Fernandes, Ricardo; Hospital de Santa Maria, Departamento da Criança e da Família; Faculdade de Medicina de Lisboa, Instituto de Medicina Molecular, Laboratorio de Farmacologia Clinica e Terapeutica<br>Scott, Shannon; University of Alberta, Nursing<br>Hartling, Lisa; University of Alberta, Pediatrics
**Primary Subject Heading**:	Paediatrics
Secondary Subject Heading:	Patient-centred medicine, Respiratory medicine, Infectious diseases
Keywords:	Community child health < PAEDIATRICS, Paediatric infectious disease & immunisation < PAEDIATRICS, RESPIRATORY MEDICINE (see Thoracic Medicine), Paediatric thoracic medicine < THORACIC MEDICINE, Respiratory infections < THORACIC MEDICINE

SCHOLARONE™
Manuscripts

For peer review only

Which outcomes are important to patients and families who have experienced pediatric acute respiratory illness? Findings from a social media engagement strategy

Michele P Dyson, Kassi Shave, Allison Gates, Ricardo M Fernandes, Shannon D Scott, Lisa Hartling

Michele P Dyson, Assistant Professor, Department of Pediatrics, University of Alberta

Kassi Shave, Former Research Assistant, Alberta Research Centre for Health Evidence (ARCHE), Department of Pediatrics, University of Alberta

Allison Gates, Research Associate, Alberta Research Centre for Health Evidence (ARCHE), Department of Pediatrics, University of Alberta

Ricardo M Fernandes, Pediatrician, Department of Pediatrics, Hospital de Santa Maria, Lisbon

Shannon D Scott, Professor, Faculty of Nursing, University of Alberta

Lisa Hartling, Professor, Department of Pediatrics, University of Alberta

Correspondence to: Dr. Michele P Dyson, 4-474 Edmonton Clinic Health Academy, University of Alberta, 11405 – 87 Avenue, Edmonton, Alberta, Canada T6G 1C9, E-mail: mdyson@ualberta.ca, Phone: 1.780.492.1241

For peer review only - http://bmjopen.bmj.com/site/about/guidelines.xhtml

ABSTRACT

Objectives. To identify the outcome priorities of parents of children who had experienced an acute respiratory infection (ARI).

Design. This was a two-phase, mixed methods study. We used a cross-sectional quantitative web-based survey to elicit parents' priorities for pediatric ARI. We then used a discussion moderated via Facebook to elucidate richer descriptions of parents' priorities.

Setting. Survey and discussion data were collected via the Internet.

Participants. 110 parents (90% female, median age 35 years, 92.7% urban dwelling, 94.5% with a post-secondary education) with a child who had experienced an ARI responded to the survey. Four parents participated in the Facebook discussion.

Primary and secondary outcome measures. The primary outcome was parents' rankings of outcomes related to pediatric ARI. The secondary outcomes were the alignment of parent-reported important outcomes with those commonly reported in Cochrane systematic reviews (SRs).

Results. Commonly reported ARIs included croup (44.5%), wheezing (43.6%), and influenza (38.2%). Parents ranked major complications, illness symptoms, and length of stay as the most important outcome categories. With respect to specific outcomes, severe complications, major side effects, doctor's assessment, relapse, oxygen supplementation, and results from lab measures were reported as most important (75th-99th percentile). Taking time off work, mild complications, interference with daily activities, treatment costs, absenteeism, follow-up visits and other costs were deemed minimally important (<25th percentile). In 35 Cochrane SRs, 29 unique outcomes were reported. Although participants' priorities sometimes aligned with outcomes frequently reported in the literature, this was not always true. Additional priorities from the survey (n=50) and Facebook discussions (n=4) included healthcare access, interacting with healthcare providers, education, impact on daily activities, and child wellbeing.

Conclusions. In the context of pediatric ARI, parents' priorities did not always align with commonly researched outcomes. Appealing and efficient strategies to engage patients and parents in research should be developed.

Key Words: social media, child health, patient-centered outcomes research, stakeholder engagement

For peer review only - http://bmjopen.bmj.com/site/about/guidelines.xhtml

STRENGTHS AND LIMITATIONS OF THIS STUDY

- The identification of patient-important outcomes is a necessary precursor to the conduct of research that is relevant to themselves and their families, though engaging patients in research is challenging.
- We used web-based tools and social media platforms to recruit and engage patients and identify patient-important outcomes for pediatric acute respiratory infections.
- Though nearly three-quarters of online adults use social media, engaging in health research online may appeal only to certain sub-populations, so the findings may not be generalizable.

For peer review only

For peer review only - http://bmjopen.bmj.com/site/about/guidelines.xhtml

INTRODUCTION

The determination of outcomes that matter to patients is foundational to the conduct of research that is relevant to them and their families. With an increasing emphasis on patient-centeredness in clinical research, numerous organizations and strategies have been established with this as their mandate (e.g., Canada's Strategy for Patient-Oriented Research [1], the Patient-Centered Outcomes Research Institute in the United States [2], and INVOLVE in the United Kingdom [3]). Involving patients in the research process will ensure that funded investigations utilize questions, outcomes, and interventions that are aligned with their needs and priorities [4-6].

There are many complexities involved in selecting outcomes, and little published guidance for investigators exists [7]. There is significant heterogeneity in the outcomes measured and reported in studies of specific diseases, which may in part occur due to uncertainty around which outcomes are patient-important [7]. The development of core outcome sets, in which a minimum group of agreed-upon outcomes is measured and reported on across clinical research in a specific condition, has been proposed as a solution to these issues [8]. Sinha et al. [7] identified 13 groups formed to develop core outcome sets for pediatric clinical trials, including the Core Outcome Measures in Effectiveness Trials (COMET) and Outcome Measures in Rheumatology (OMERACT). The COMET initiative was launched in 2010 to bring together individuals interested in developing core outcome sets, and to collate outcome sets and relevant resources [8]. Established in 1992, OMERACT is a consensus initiative that has developed a number of widely utilized core outcome sets for rheumatologic conditions, with patients actively involved in the process since 2002 [9, 10].

One criticism of commonly used methods to develop core outcome sets is that they do not include a systematic survey of stakeholders [9]. Social media represents a medium where patients and their caregivers increasingly interact online [11, 12], providing an opportune channel for engagement in the development of core outcomes. Nearly three quarters (74%) of online adults use social media, with Facebook continuing to be the most popular social media site, and multi-platform use increasing in prevalence [13, 14]. Despite the global pervasiveness of social media, its use for engaging patients and/or caregivers in the outcome selection process has not extensively been explored.

We conducted a two-phase mixed methods study using social media to recruit and engage the parents of children with an acute respiratory infection (ARI) to elucidate patient-important outcomes. Acute

For peer review only - http://bmjopen.bmj.com/site/about/guidelines.xhtml

respiratory infections are common among children, represent a significant source of morbidity, and are one of the leading causes of illness, emergency department visits, and hospitalization [15, 16]. There is a scarcity of research on patient perspectives in this area [7]. Using social media, we aimed to: (a) recruit and survey parents to identify their priorities for ARI outcomes as compared to those commonly reported in the literature, and (b) engage parents in discussions to elucidate the rationale for their priorities, as identified in the quantitative survey.

METHODS

This mixed methods study utilized an explanatory sequential design [17], and involved two phases: 1) a quantitative survey to determine parent priorities for ARI outcomes; 2) a qualitative follow-up in which we sought elaboration on parents' priorities. Institutional ethics approval was obtained from the University of Alberta Research Ethics Board. Parents and/or caregivers were eligible to participate if they had a child aged 0-17 years who had experienced one or more episodes of acute asthma, bronchiolitis, croup, influenza, pharyngitis/tonsillitis, pneumonia, sinusitis, and/or wheezing. Consent was provided through overt action by completing the survey or publishing responses online. A process evaluation of our social media strategy is reported elsewhere [18].

Phase I: Quantitative Survey

Development and Pre-Testing

Survey development was informed by previous outcome-related instruments [19], outcomes measured in the literature, and input from content experts and consumers. For each of the conditions of interest, we identified systematic reviews (SRs) published up to 2013 from the Cochrane Database of Systematic Reviews (n=35) and their included studies to determine which outcomes have been measured and reported. Outcomes were grouped into a broad classification system with seven categories (disease activity; disease complications; adverse effects of therapy; functional status; social and family outcomes, including quality of life; long-term effects of interventions; and resource utilization), based on a SR of studies conducted to determine which outcomes to measure in children [7, 19, 20]. Three outcomes identified as relevant by content experts and consumers during pilot testing were also incorporated to form a finalized list of ten categories. These included: major complications; symptoms; length of stay in the emergency department or hospital; needing to see a doctor; returns visits to a doctor or the hospital; reactions to medications; medical test results; maintenance of day-to-day activities; minor complications; and cost of illness.

For peer review only - http://bmjopen.bmj.com/site/about/guidelines.xhtml

We ensured survey accessibility across different operating systems, including touch screen (e.g., tablets, smartphones) or keyboard (e.g., desktop computers) technology. We wrote the materials at a sixth grade reading level and conducted pilot testing with 8 parents and 10 researchers/clinicians to ensure that the language and flow of questions were appropriate.

Recruitment

We created an online and social media presence via a study website (www.outch-study.com), a Facebook page (OUTCH), and a Twitter account (@OUTCH_Study). We used snowball sampling [21] to recruit parents. First, we focused on identifying and engaging recruitment targets with the potential for a high yield of participants. We then expanded our scope through referrals and diffusion via social media. Tactica Interactive (http://tactica.ca), a digital media enterprise, was hired to broaden our sampling frame via a Facebook advertising strategy.

We collaborated with organizations interested in ARI and patient engagement to advertise our research via websites and other channels: The Alberta Centre for Child, Family & Community Research (ACCFCR, now known as PolicyWise for Children and Families; a provincial organization linking government, academia, and the community in a focus on evidence-informed policy and practice) [22], TRanslating Emergency Knowledge for Kids (TREKK; a national network of researchers and clinicians invested in improving pediatric emergency care) [23]; the Cochrane Consumer Network (CCNet; an international network of healthcare consumers with an interest in evidence-based medicine) [24]; and the Stollery Family Centered Care Network (a local children's hospital-based network of patients and families that provide input into patient care) [25]. We also engaged an online (Facebook and Twitter) parenting community, Mommy Connections, that regularly promoted the study through their networks.

Data Collection

The quantitative survey was administered by Nooro Online Research (https://nooro.com/index.html) for 14 weeks from December 2013 to March 2014. Links to the survey were provided through the study website, Facebook, and Twitter accounts and were completed anonymously. The only identifying information was an optional e-mail address for entry into a prize draw.

The survey included a combination of open- and closed-ended questions to determine the relative importance of outcomes currently measured in trials and SRs of ARI in children. The outcome categories

For peer review only - http://bmjopen.bmj.com/site/about/guidelines.xhtml

were presented and parents were asked to identify their top five priorities from the list. Then, individual outcomes were presented and parents were asked to indicate their importance using a digital sliding scale, conceptually similar to a visual analog scale. The two ends of the scale were anchored with the statements ‘Not important [/concerning] at all’ and ‘Extremely important [/concerning].’ Parents were also asked to indicate additional items that were considered important to patients and their families, but may not have been addressed in the literature. The survey platform was also used to collect demographic data.

Phase II: Qualitative Follow-Up

In the second phase, we conducted an Internet-based, descriptive qualitative study [26] to interact with parents and elicit elaboration on their perceptions of the importance of ARI outcomes. This study occurred across an eight-week period from January to March 2016 during which parents were engaged in discussion through an open online focus group hosted on the study Facebook page.

Recruitment

To recruit parents, we utilized a snowball sampling technique [21]. We first asked potentially high yield sources of participants to promote our study, including local and national online parenting communities (n=16), children’s hospitals and associated foundations (n=14), and patient groups (n=3) with access to a large consumer audience. We also asked individuals and organizations within our existing networks to promote the study. Recruitment occurred throughout the study period, and was almost exclusively carried out through Facebook. Some organizations or individuals promoted the study on Twitter or a blog, but all links posted drove user traffic back to the study Facebook page.

One variable of interest in this study was the reach of social media as a recruitment strategy (reported elsewhere [18]); therefore, the sample size was an outcome, rather than a pre-defined condition. To accommodate this, we did not define the number of participants *a priori*, instead allowing the detail that emerged from our data collection to guide the extent of recruitment. However, we did aim to recruit a sample size as guided by the principles of data saturation, in which data would be collected until no new themes emerged [27].

Data Collection

For peer review only - http://bmjopen.bmj.com/site/about/guidelines.xhtml

Throughout the study period, each week had a discussion theme modelled after the structure of the survey, and posts were published daily covering varying aspects of this theme. Three different types of posts were published: promotional posts prior to the study launch; parent-friendly content about ARIs; and discussion questions where parents were encouraged to share their thoughts and experiences. The online focus group was moderated by two members of the study team (MPD, KS) and all posts by participants were followed up promptly with a response. An interview guide (Supplementary File 1) was developed to guide weekly topics and for reference during discussion moderation.

Data Analysis

Quantitative Analysis

Survey data were analyzed using SPSS (v. 22.0, IBM Corporation, Armonk, NY) and described using descriptive statistics (mean±SD; rank order). To compare the published literature to the patient-important outcomes identified by parents, we grouped the outcomes identified in the Cochrane SRs into percentile ranges based on the number of SRs in which each was reported ('frequently reported': 75th-99th percentile; 'moderately reported': 25th-74.9^{th} percentile; and 'infrequently reported': <25th percentile). Parents' rankings of the importance of outcomes were grouped similarly ('most important': 75th-99th percentile; 'moderately important': 25th-74.9^{th} percentile; and 'least important': <25th percentile), and all comparisons were strictly descriptive. Parents who had healthcare insurance were self-identified in the survey and excluded from all analyses related to healthcare costs as these may not hold relevance.

Qualitative Analysis

Content posted by participants during the focus groups was extracted verbatim to form transcripts. These, along with the open-ended responses to the survey, were imported into NVivo 10 (QSR International, Melbourne, Australia) data management software. Data were analyzed inductively for themes. Two investigators (MPD, KS) participated in coding, following a three stage process: 1) reading through the data, making notes on themes and significance that were then compiled into a preliminary version of the codebook; 2) rereading the data and coding using the concepts identified in the first phase; and 3) refining and applying the codes to the text upon a third review [28]. Coders met to discuss progress and reach consensus on differing interpretations. Data collection and analysis occurred concurrently, following an iterative process to monitor progress and allow for follow up on ideas as they emerged.

For peer review only - http://bmjopen.bmj.com/site/about/guidelines.xhtml

RESULTS

Demographic Characteristics

A total of 110 people responded to the survey (Table 1). The survey website received 5,207 visits, a view rate (i.e., the ratio of unique survey visitors/unique site visitors) of 4.1% (205/5,207), and a completion rate (i.e., the ratio of unique visitors who completed the survey/users who agreed to participate) of 53.7% (110/205).

Ninety percent (n=99) of respondents were female. The median age was 35 years, and 89% (n=97) of respondents had received a college/university or post-graduate education. Most survey respondents were married (n=98; 89%), urban-dwelling (n=102; 93%), and resided in Canada (n=77; 70%). More than half of respondents reported an annual household income of >$90,000 USD (n= 69; 63%). Respondents were predominantly parents (n=106; 96%), and had a median of two children in the home (range 0-4). The respondents' children most commonly experienced croup (n=49; 45%), wheezing (n=48; 44%), and influenza (n=42; 38%). The most concerning ARIs were croup (n=20; 25%), pneumonia (n=16; 20%), and asthma (n=15; 19%). The median year that the ARI occurred was 2012 (range 1994-2013), when the child was 1-year-old (range <1 month-10 years old). Most children did not have a chronic illness (n=90; 82%) and did not experience a hospital admission due to this ARI (n=84; 80%).

Table 1. Survey participant demographics (N=110)

**Characteristic**	**N (%)**
Gender	Female: 99 (90.0)<br>Male: 11 (10.0)
Age (years) (median (range))	35 (18-67)
Highest level of education	Some high school: 1 (0.9)<br>High school graduate: 5 (4.6)<br>Some college/university: 7 (6.4)<br>College/university graduate: 50 (45.5)<br>Post-graduate education/degree: 47 (42.7)
Marital status	Single: 5 (4.6)<br>Married/Common-law: 98 (89.1)<br>Separated/divorced/widowed: 7 (6.4)
Annual household income (USD$)	<30,000: 5 (4.6)<br>30-49,999: 7 (6.4)<br>50-69,999: 15 (13.6)<br>70-89,999: 14 (12.7)<br>>90,000: 69 (62.7)

For peer review only

Characteristic	N (%)
Country of residence	Australia: 2 (1.8)<br>Canada: 77 (70.0)<br>England: 8 (7.3)<br>India: 2 (1.8)<br>Portugal: 2 (1.8)<br>United States: 19 (17.3)
Type of community	Urban (≥10,000): 102 (92.7)<br>Rural (<10,000): 7(6.4)<br>Missing: 1 (0.9)
Number of children in home (median (range))	2 (0-4)
Relationship to child	Parent: 106 (96.4)<br>Step-parent: 0 (0)<br>Grandparent: 4 (3.6)<br>Other: 2 (1.8)
Type of ARI	Bronchiolitis: 29 (26.4)<br>Croup: 49 (44.6)<br>Strep throat/tonsillitis: 36 (32.7)<br>Wheezing: 48 (43.6)<br>Influenza: 42 (38.2)<br>Pneumonia: 24 (21.8)<br>Asthma: 29 (26.4)<br>Other: 23 (20.9)
Most concerning ARI	Bronchiolitis: 10 (12)<br>Croup: 20 (25)<br>Strep throat/tonsillitis: 3 (4)<br>Wheezing: 6 (7)<br>Influenza: 2 (3)<br>Pneumonia: 16 (20)<br>Asthma: 15 (19)<br>Other: 6 (7)
Year of ARI (median (range))	2012 (1994-2013)
Child age at time of ARI (median (range))	1 year (<1 month-10 years)
Hospital admissions due to ARI	Yes: 21 (20)<br>No: 84 (80)
Chronic illness	Yes: 20 (18)<br>No: 90 (82)

ARI: Acute Respiratory Infection

Quantitative Outcome Rankings

The overall ranking of categorized outcomes is shown in Table 2. On average, parents ranked major complications from the child's illness (e.g., long-term disability), illness symptoms (e.g., coughing, fever, sore throat), and length of stay in the emergency department or hospital as the most important

For peer review only - http://bmjopen.bmj.com/site/about/guidelines.xhtml
For peer review only

outcome categories. Of least importance were the costs of their child's illness (e.g., medicine or child care), minor complications from the child's illness (e.g., cough or rash), and maintenance of day-to-day activities. The overall ranking of individual outcomes revealed that parents were most concerned about severe complications (mean score on a scale from 1-100: 94.5), major side effects (86.7), and their doctor's assessment (83.9). Other costs (e.g., child care, parking, lost income) (31.3), scheduled follow-up visits (38.1), and school/daycare absenteeism (40.4) received the lowest mean scores. When parents were grouped according to ARI their child had experienced and their rankings of the importance of outcomes were compared, Spearman correlation coefficients revealed strong agreement, indicating that perceptions of importance were consistent across conditions (Table 3).

Table 2. Overall ranking of categorized items (N=110)

Rank Order[a]	Category
**1**	Major complications from child's illness (e.g., long-term disability)
**2**	Illness symptoms (e.g., coughing, fever, sore throat)
**3**	Length of stay in the emergency department or hospital
**4**	Child needing to see a doctor
**5**	Return visits to the doctor or hospital
**6**	Child's reaction to his or her medicine (e.g., side effects)
**7**	Child's medical test results
**8**	Maintenance of day-to-day activities
**9**	Minor complications from child's illness (e.g., cough or rash)
**10**	Costs of child's illness (e.g., medicine or child care)

[a]Ordered from most to least important to parents.

Table 3. Agreement between acute respiratory infection type and parent ranking of categorized outcomes (N=110)

Type of Acute Respiratory Infection	Spearman Correlation Coefficient*
Bronchiolitis	0.94
Croup	0.75
Strep Throat/Tonsillitis	0.66
Sinusitis	0.87
Wheezing	0.76
Influenza	0.66
Pneumonia	0.85
Asthma	0.67
Other	0.50

*0-0.2: poor/slight agreement; 0.2-0.4: fair agreement; 0.4-0.6: moderate agreement; 0.6-0.8: substantial agreement; 0.8-1: near perfect agreement

For peer review only - http://bmjopen.bmj.com/site/about/guidelines.xhtml

A comparison of the outcomes reported in the literature to those reported as important by parents is shown in Table 4. The 35 Cochrane SRs reported a total of 221 outcomes, of which 29 were unique (reported in 1- 26 SRs). Adverse events were the most frequently measured outcome (f=26; 11.8%) in our sample of Cochrane SRs, and similarly, severe complications (score: 94.5/100) and major side effects (86.7) were ranked as the most important (75th-99th percentile) to parents. Likewise, parents ranked returning to school/work and the cost of treatment as least important (40.4 and 44, respectively; <25th percentile), and these outcomes were infrequently measured in Cochrane SRs (f=2; <25th percentile).

There were many discrepancies between the outcomes measured in Cochrane SRs and the outcomes parents ranked as important. Relapse and the need for oxygen supplementation were ranked among the most important outcomes by parents (81.8 and 81.6, respectively; 75th-99th percentile), but were measured moderately frequently (f=8 and f=5, respectively; 25th-74.9^{th} percentile) in Cochrane SRs. Similarly, the results from lab measures were ranked highly by parents (81.4; 75th-99th percentile), but were infrequently reported in Cochrane SRs (f=4; 25th-74.9^{th} percentile). The need for medication was one of the most frequently reported outcomes in Cochrane reviews (f=13; 75th-99th percentile), but was ranked as moderately important by parents (55.1; 25th-74.9^{th} percentile). Admission rate was the second most frequently measured outcome in Cochrane SRs (f=19; 75th-99th percentile), yet was also ranked less favorably among parents (76.9; 25th-74.9^{th} percentile). While the doctor's assessment of how the child is doing was ranked as the third most important outcome (83.9; 75th-99th percentile) by parents, only clinical scores/symptom scores, one of three corresponding components of this outcome (clinical scores/symptom scores; patient improvement; observed response to treatment) was measured frequently (f=15; 75th-99th percentile) in Cochrane SRs. Patient improvement was measured moderately frequently (f=6; 25th-74.9^{th} percentile), and observed response to treatment was measured the least frequently of any outcome included in this study (f=1; <25th percentile).

Table 4. Parent ranking of individual outcomes compared to frequency of measurement in Cochrane systematic reviews

Outcomes reported in Cochrane systematic reviews (N=35)	Frequency of reporting	Parent ranking of importance of outcomes (N=110)	Mean Score ±SD (/100)
**75th-99th percentile**			
Adverse events (local and systemic)	26	Severe complications	94.5 ±14.5
Admission rate (hospital, ED, ICU)	19	Major side effect	86.7 ±18.9

For peer review only - http://bmjopen.bmj.com/site/about/guidelines.xhtml

Physical signs	18	Doctor's assessment	83.9 ±19.6
Clinical measures	17	Relapse	81.8 ±18.4
Clinical scores/symptom scores	15	Oxygen supplementation	81.6 ±21.6
Length of stay/time to discharge (hospital, ED, ICU)	15	Results from lab measures	81.4 ±19.4
Need for medication	13		
**25th-74.9^{th} percentile**			
Severity of symptoms	12	Length of hospital stay	78.6 ±22.1
Duration of symptoms	10	Trip to emergency department	77.6 ±23.9
Complications	9	Time to recovery	77.4 ±17.8
Rates of relapse	8	Hospital admission	76.9 ±23.7
Clinical treatment failure	7	Return healthcare visit	76.9 ±24.0
Patient improvement	6	Not eating/drinking well	65.9 ±22.8
Time to resolution of illness/time to recovery	6	Lack of sleep	63.2 ±20.0
Duration of oxygen supplementation	5	Length of stay in emergency department	62.4 ±26.1
Mortality	5	Minor side effect	55.1 ±24.8
Laboratory measures	4	Prescription for medication	55.1 ±25.6
Readmission	4	Appointment with GP/pediatrician	48.4 ±27.8
		Arranging child care	48.3 ±30.1
**<25th percentile**			
Clinical cure	3	Taking time off work	47.2 ±28.4
Compliance and tolerance	3	Mild complications	46.3 ±23.3
Quality of life/patients' well-being	3	Interference with daily activities	44.6 ±23.5
Return healthcare visits	3	Treatment costs	44.0 ±32.5
Return to school/work	2	School/daycare absenteeism	40.4 ±26.5
GP visits	2	Scheduled follow-up visits	38.1 ±24.8
Treatment cost	2	Other costs	31.3 ±31.3
Adverse events that necessitated discontinuation of treatment	1		
Sleep disturbance	1		
Parental perception of child's status	1		
Observed response to treatment	1		

Qualitative Synthesis

A total of 50 respondents provided qualitative responses on the survey. Four participants contributed to the discussion on the Facebook page. The thematic analysis revealed five main analytical themes relating to parents' priorities and concerns when their child had an ARI: 'accessing health care'; 'interacting with healthcare providers'; 'illness education'; 'impact of illness on daily activities'; and 'child wellbeing'.

Accessing Health Care

Though not expressed by all parents, having timely access to health care for their child was a primary concern for many. Being able to get the medical advice they needed, without encountering substantial

For peer review only

For peer review only - http://bmjopen.bmj.com/site/about/guidelines.xhtml

waiting times was important. Parents expressed concern about recognizing the signs and symptoms of their child's disease process. Determining whether their child's symptoms were serious or minor was considered challenging. Parents lacked confidence in deciding when to seek medical attention, particularly when the child frequently experienced breathing problems. Overall, parents shared wanting to avoid making unnecessary health care visits, and expressed the importance of receiving helpful tips from health care providers for managing acute symptoms at home.

Interacting with Health Care Providers

When parents accessed health care for their child, they desired to be taken seriously by healthcare providers. Parents described concern about their child's healthcare provider being dismissive or uninterested in their chief complaints about their child's health. Strong communication with healthcare providers was widely valued by parents. Parents expressed wanting to feel heard, and to have a medical team that was both helpful and thorough in explaining their findings in a way that was direct and understandable.

Illness Education

Receiving education about their child's illness was widely regarded as important. Parents described requiring a complete understanding of what to expect, when to seek medical attention, and what the recovery time and process would be like. Information about the long-term impact of their child's health condition, and what effect recurrences or exacerbations might have, was regarded as of specific importance. When appropriate, parents regarded being presented with a variety of treatment options as critical. Parents expressed wanting to be involved in their child's recovery, and regarded education about their child's illness as essential to taking an active role.

Impact of Illness on Daily Activities

Parents described concern around interruption of their work schedules and sleeping routines. Being able to stay at home or in hospital with their child when they were unwell was important to parents, while not always possible when balancing financial and care responsibilities for other siblings. Disruption of sleep routines for parents and siblings was also described as concerning, particularly among parents of children with uncontrolled cough. Potential spread of their child's ARI between siblings and to the parents themselves was noted as a primary concern, and highly disruptive to daily activities.

For peer review only - http://bmjopen.bmj.com/site/about/guidelines.xhtml

Child Wellbeing

Parents shared their concern for their child's psychological wellbeing when they had an ARI. Concern about how their child was coping when they were unwell, particularly for those children with recurring or chronic ARI, was emphasized. Parents also expressed concern about how others treated their child when they were sick. One parent described concern over their child being treated like an 'invalid' when experiencing an acute asthma exacerbation.

DISCUSSION

Knowledge of patients' and their families' priorities is essential to guide the conduct of research that is relevant to themselves as well as to clinicians and policymakers [19]. Using social media, we engaged >100 parents over 14 weeks in a survey to elucidate the outcomes that they deemed most important with regard to pediatric ARIs. Parents' most important concerns included clinical outcomes like major complications, symptoms, and length of stay in the emergency department or hospital. Psychosocial outcomes, and the ability of the family to cope during a child's illness, were also important. Parents were less concerned with mild complications, interference with daily activities, and the cost of the illness.

Our findings reinforce the growing recognition that insufficient consideration is being paid to the selection of outcomes within clinical trials [29]. Not only did our survey of 35 Cochrane SRs reveal that a diverse array of outcomes has been reported across trials of pediatric ARIs, but the most commonly reported outcomes were not always aligned with those that parents considered important. For example, some chief concerns such as relapse and the long-term consequences of illness were rarely or never described in the literature. When asked to elaborate on their priorities, parents cited psychosocial outcomes including their child's overall wellbeing and ability to cope with their illness (e.g., ability to eat and drink, sleep quality), stigma associated with their child's illness or being treated differently by their peers, and their own knowledge and ability to play an active role in their child's care as very important. Meanwhile, the bulk of the research in child health has focused on exploring, measuring, and improving upon biological outcomes, with relatively little attention being paid the psychosocial impact of illness [20, 30, 31].

Health, though difficult to define, encompasses not only an individual's physical condition, but also their emotional, and psychological wellbeing [32]. The ability of a child to adapt to their illness, an integral

For peer review only - http://bmjopen.bmj.com/site/about/guidelines.xhtml
For peer review only

component of overall health [32] and a point of great concern for parents, requires more attention in child health research. The need for the development of core outcome sets [33], and especially core outcome sets that incorporate patient-reported and patient-centered outcomes [34] has garnered increased attention in recent years. More consistent reporting of outcomes for pediatric ARI will be necessary to facilitate evidence synthesis [29, 34], and to enhance trustworthiness by reducing the risk of reporting bias [35]. Focusing investigations and interventions on outcomes that are important to patients, their families, and other stakeholders will also contribute to reducing research waste [36].

Moving toward greater inclusion of patient-important outcomes in pediatric health research is challenged by the fact that children and their parents can be difficult to reach and engage. Given the pervasiveness of social media use via multiple platforms by patients and their caregivers [37], we postulated that this would provide an opportune medium to learn parents' perspectives. Though we experienced relative success in recruiting parents to complete the survey, qualitative engagement via the Facebook discussion group was more arduous. Moreover, despite moderate success in engaging parents, we did not gather any information from children themselves. Children have the right to participate in matters that affect their own lives [38], and can provide unique perspectives that cannot be elicited from their caregivers. Nevertheless, children also require protection, and the extent to which minors can understand and express their own healthcare needs remains controversial [38].

The challenges that we experienced are not unique. A review of studies that addressed the process of outcome selection identified only three studies that involved parents and none that involved children in the identification of pediatric patient-important outcomes [7]. In deciding which outcomes should be measured in pediatric ARI, it will be essential that stakeholders with varied perspectives, including parents, children, researchers, clinicians, and decision-makers convene and reach agreement on research priorities [29]. Suggested approaches like the Delphi technique and nominal group technique provide a means for stakeholders to reach unanimity on important outcomes in child health research [7, 29]. These are, however, time consuming, resource-intensive and are highly burdensome to participants, which may limit recruitment and engagement. Further guidance is required on consensus methods that are efficient and appealing to patients, families and other stakeholders. Methods that are understandable to children will need to be developed if researchers are to uphold the rights of minors to be involved in their own healthcare [38]. Reconciling children's and parents' perspectives, and the extent to which minors should be involved in the consensus process, requires further study.

For peer review only - http://bmjopen.bmj.com/site/about/guidelines.xhtml
For peer review only

Limitations

Our sample of parents and guardians were highly educated, many of whom had family incomes well above the national median, were mostly urban dwelling, and mainly Canadian, limiting the generalizability of the findings. For example, participants with lower incomes, or those residing in countries without publicly funded health care may have placed more importance on the cost of illness. Because we had difficulty engaging parents in the qualitative discussion, and did not reach saturation, the emergent themes should be interpreted as preliminary.

CONCLUSIONS

The conduct and reporting of research of little relevance to the primary stakeholders represents a significant source of research waste, and appears prevalent in the context of pediatric ARI. The development of core outcome sets that include patient-important outcomes will facilitate evidence synthesis and reduce reporting bias, supporting the utility and trustworthiness of research findings. Future investigations are required to elucidate ways to make engagement in research more efficient and appealing to patients and their families.

ACKNOWLEDGEMENTS

The authors would like to thank Sarah Oberik-Olivieri for her graphic design work for this study.

COMPETING INTERESTS

All authors have completed the ICMJE uniform disclosure form at www.icmje.org/coi_disclosure.pdf and declare: no support from any organisations for the submitted work; no financial relationships with any organisations that might have an interest in the submitted work in the previous three years; no other relationships or activities that could appear to have influenced the submitted work.

FUNDING

This work was supported by KT Canada, grant number CIHR 87776 (SG-1) and Alberta Innovates Health Solutions, grant number 201400561. LH is supported in part by a Canadian Institutes of Health Research New Investigator Award. SS is a Canada Research Chair (Tier II) for Knowledge Translation in Child Health and is also supported by an Alberta Innovates Health Solutions Population Health Investigator Award.

For peer review only - http://bmjopen.bmj.com/site/about/guidelines.xhtml

The funders had no role in study design, data collection and analysis, decision to publish, or preparation of the manuscript.

AUTHOR CONTRIBUTIONS

MPD and LH designed the study. MPD oversaw all aspects of the study's implementation. MPD and KS collected, analysed and interpreted the data with input from AG, LH, RMF, and SDS. All authors had full access to the data, and can take responsibility for the integrity of the data and the accuracy of the data analysis. KS and AG drafted the manuscript. MPD, LH, RMF and SDS reviewed the manuscript critically for intellectual content. All authors approved the version of the manuscript that was submitted to the journal. MPD affirms that the manuscript is an honest, accurate, and transparent account of the study being reported; that no important aspects of the study have been omitted; and that any discrepancies from the study as planned have been explained.

For peer review only

For peer review only - http://bmjopen.bmj.com/site/about/guidelines.xhtml

REFERENCES

1. Canadian Institutes of Health Research [Internet]. Strategy for Patient-Oriented Research. 2016 [cited 2017 May 1]. Available from: http://www.cihr-irsc.gc.ca/e/41204.html.
2. Patient-Centered Outcomes Research Institute [Internet]. 2016 [cited 2017 May 1]. Available from: http://www.pcori.org/.
3. National Institute for Health Research [Internet]. INVOLVE. 2015 [cited 2017 May 1]. Available from: www.invo.org.uk.
4. Frank L, Basch E, Selby JV. Patient-Centered Outcomes Research Institute. The PCORI perspective on patient-centered outcomes research. *JAMA* 2014;312(15):1513-4.
5. National Institute for Health Research [Internet]. INVOLVE. Exploring the impact of public involvement on the quality of research: examples. 2013 [cited 2017 May 1]. Available from: http://www.invo.org.uk/wp-content/uploads/2013/08/invoNETexamples2013.pdf.
6. Methodology Committee of the Patient-Centered Outcomes Research Institute (PCORI). Methodological standards and patient-centeredness in comparative effectiveness research: the PCORI perspective. *JAMA* 2012;307(15):1636-40.
7. Sinha I, Jones L, Smyth RL, et al. A systematic review of studies that aim to determine which outcomes to measure in clinical trials in children. *PLoS Med* 2008;5(4):e96.
8. COMET Initiative [Internet]. 2017 [cited 2017 May 1]. Available from: http://www.comet-initiative.org/.
9. Boers M, Kirwan JR, Wells G, et al. Developing core outcome measurement sets for clinical trials: OMERACT filter 2.0. *J Clin Epidemiol* 2014;67(7):745-53.
10. Boers M, Brooks P, Strand CV, et al. The OMERACT filter for Outcome Measures in Rheumatology. *J Rheumatol* 1998;25(2):198-9.
11. Fernandez-Luque L, Bau T. Health and social media: perfect storm of information. *Healthc Inform Res* 2015;21(2):67-73.
12. Fox S, Duggan M. Health Online 2013 [Internet]. 2013 [cited 2017 May 1]. Available from: http://www.pewinternet.org/files/old-media/Files/Reports/PIP_HealthOnline.pdf.
13. Duggan M, Ellison NB, Lampe C, Lenhart A, Madden M. Social Media Update 2014 [Internet]. 2015 [cited 2017 May 1]. Available from: http://www.pewinternet.org/2015/01/09/social-media-update-2014/.
14. Facebook [Internet]. Stats. 2017. [cited 2017 May 1]. Available from: http://newsroom.fb.com/company-info/.

For peer review only - http://bmjopen.bmj.com/site/about/guidelines.xhtml

15. Doan Q, Enarson P, Kissoon N, et al. Rapid viral diagnosis for acute febrile respiratory illness in children in the Emergency Department. *Cochrane Database Syst Rev* 2014;(9):CD006452.

16. Silka PA, Geiderman JM, Goldberg JB, et al. Demand on ED resources during periods of widespread influenza activity. *Am J Emerg Med* 2003;21(7):534-9.

17. Creswell JW, Plano Clark VL. Designing and conducting mixed methods research. Thousand Oaks, CA: Sage Publications; 2007.

18. Dyson MP, Shave K, Fernandes RM, et al. Outcomes in child health: Exploring the use of social media to engage parents in patient-centered outcomes research. *J Med Internet Res* 2017;19(3):e78.

19. Sinha IP, Gallagher R, Williamson PR, et al. Development of a core outcome set for clinical trials in childhood asthma: a survey of clinicians, parents, and young people. *Trials* 2012;13:103.

20. Sinha IP, Altman DG, Beresford MW, et al. Standard 5: selection, measurement, and reporting of outcomes in clinical trials in children. *Pediatrics* 2012;129 Suppl 3:S146-52.

21. Miles MB, Huberman AM. Qualitative data analysis: An expanded sourcebook. Thousand Oaks, CA: Sage Publications;1994.

22. PolicyWise for Children and Families [Internet]. About Us. 2017 [cited 2017 May 1]. Available from: https://policywise.com/about-us/.

23. TRanslating Emergency Knowledge for Kids (TREKK) [Internet]. About Us. 2017 [cited 2017 May 1]. Available from: http://trekk.ca/about.

24. Cochrane. Cochrane Consumer Network. 2017 [cited 2017 May 1]. Available from: http://consumers.cochrane.org/.

25. Alberta Health Services [Internet]. Family Centred Care (FCC). Stollery Children's Hospital. 2017 [cited 2017 May 1]. Available from: http://www.albertahealthservices.ca/services/page10093.aspx.

26. Sandelowski M. What's in a name? Qualitative description revisited. *Res Nurs Health* 2010;33(1):77-84.

27. Guest G, Bunce A, Johnson L. How many interviews are enough? An experiment with data saturation and variability. *Field Methods* 2006;18(1):59-82.

28. Guest G, MacQueen KM, Namey EE. Applied thematic analysis. Thousand Oaks, CA: Sage Publications; 2012.

29. Williamson PR, Altman DG, Blazeby JM, et al. Developing core outcome sets for clinical trials: issues to consider. *Trials* 2012;13:132.

For peer review only - http://bmjopen.bmj.com/site/about/guidelines.xhtml

30. Clarke SA, Eiser C. The measurement of health-related quality of life (QOL) in paediatric clinical trials: a systematic review. *Health Qual Life Outcomes* 2004;2:66.

31. Sinha IP, Williamson PR, Smyth RL. Outcomes in clinical trials of inhaled corticosteroids for children with asthma are narrowly focussed on short term disease activity. *PLoS One* 2009;4(7):e6276.

32. Huber M, Knottnerus JA, Green L, et al. How should we define health? *BMJ* 2011;343:d4163.

33. Gargon E, Williamson PR, Altman DG, et al. The COMET initiative database: progress and activities update (2014). *Trials* 2015;16:515.

34. Macefield RC, Jacobs M, Korfage IJ, et al. Developing core outcomes sets: methods for identifying and including patient-reported outcomes (PROs). *Trials* 2014;15:49.

35. Dwan K, Gamble C, Williamson PR, et al. Systematic review of the empirical evidence of study publication bias and outcome reporting bias - an updated review. *PLoS One* 2013;8(7):e66844.

36. Chalmers I, Bracken MB, Cjulbegovic B, et al. How to increase value and reduce waste when research priorities are set. *Lancet* 2014;383(9912):156-65.

37. Hamm MP, Chisholm A, Shulhan J, et al. Social media use among patients and caregivers: a scoping review. *BMJ Open* 2013;3(5):e002819.

38. Söderbäck M, Coyne I, Harder M. The importance of including both a child perspective and the child's perspective within health care settings to provide truly child-centred care. *J Child Health Care* 2011;15(2):99-106.

For peer review only

For peer review only - http://bmjopen.bmj.com/site/about/guidelines.xhtml

Supplementary File 1

OUTCH Discussion Guide

Important Considerations

1. Is there agreement on the outcomes chosen, both among parents involved in the discussion, and between the discussion and the survey?
2. Are there areas (i.e., in current research) that have been overlooked?
3. What is the rationale for outcome selection?
4. What are considered to be clinically important or meaningful differences in the outcomes selected?

Week 1: February 1 – 5, 2016

Theme: **Getting Medical Help**

Sometimes when children are sick and their breathing is affected, they need help from a doctor.

1. Tell us about a time you needed to bring your child to a doctor because their breathing was affected.
    a. Did you have to go to see a doctor? Go to the emergency department?
    b. How did you feel?
    c. Did, or would, one have more impact on you than the other?
2. Tell us about what you find most concerning about getting medical help for your child when they are sick and their breathing is affected.
    a. Possible considerations (as prompts):
        i. how long you had to stay at the emergency department;
        ii. how long you had to stay in the hospital;
        iii. if you had to go for a scheduled follow-up visit to your family doctor or pediatrician;
        iv. if you had to go back to see a doctor after the first time because your child wasn't getting better.

- Thanks for checking out the OUTCH Study! We want to make sure that your child's health care is based on what's most important to you when their breathing is affected. This week, tell us what matters when your child needs to see the doctor. [attach graphic]

- Hey Parents! Have you had to take your child to the doctor or emergency department because they were having a hard time breathing? What was important to you?

- Excited to get this discussion going about what is important to you when your child is having a hard time breathing! Check out our page and share your experiences in the comments section.

Week 2: February 8 – 12, 2016

Theme: **Medical Tests**

Sometimes children need tests and treatment when they are sick.

For peer review only - http://bmjopen.bmj.com/site/about/guidelines.xhtml

Supplementary File 1

1. Tell us about a time your child needed tests because they were sick and their breathing was affected.
    a. What kind of tests did they need?
    b. At the time, how much did the results of things like x-rays or blood tests matter to you?
    c. How much did the doctor's assessment of your child's illness matter to you?
    d. Did, and why did, one type of assessment matter more to you than the other?

- Welcome to another week of the OUTCH Study! Help us understand your story. Tell us in the comments below about what matters to you when your child needs medical tests because they are having a hard time breathing. [attach graphic]

- If your child has needed tests like x-rays or blood tests when they're sick, what has mattered to you? Tell us in the comments below!

- Get to Know OUTCH!
We're asking you to share your stories with us, so we thought we'd tell you a little more about our team!

What does OUTCH stand for?
OUTCH stands for Outcomes in Child Health.

Who are we?
We're a group of researchers from the Department of Pediatrics at the University of Alberta, and we're the ones behind the scenes at the OUTCH Study.

What is the OUTCH Study all about?
We're interested in learning about outcomes that matter to you when your children's breathing is affected. This could mean that they have, or had, an illness like bronchiolitis, croup, strep throat or tonsillitis, a sinus infection, wheezing, the flu, pneumonia, or acute asthma. We want to talk to parents like you to make sure that our research asks the right questions, so we can improve healthcare for children and their families.

How can you help?
Tell us what matters to you in the comments section of our Facebook posts – we'd love to hear from you!

<u>Week 3: February 16 – 19, 2016 (*Family Day on February 15)</u>

Theme: **Medical Treatment**

Sometimes when children are sick, they need to take medicine or be treated in other ways.

For peer review only - http://bmjopen.bmj.com/site/about/guidelines.xhtml

Supplementary File 1

1. Tell us about a time when your child was sick and they needed treatment because their breathing was affected.
    a. What kind of treatment did they need?
        i. Were they given medicine? Oxygen?
2. How concerned were you about your child needing treatment?
    a. What was most concerning about your child needing treatment because their breathing was affected?
3. If your child has had more than one kind of treatment, have you found certain treatments more concerning than others?
4. How concerned would you be concerned about a mild side effect from the treatment or medicine, like vomiting, a rash, or diarrhea?
5. How concerned would you be about a major side effect from the treatment or medicine (like being very agitated or having a serious allergic reaction), that was bad enough that your child had to stop taking it?

- Welcome back parents! Here in Alberta we're just coming off of our Family Day long weekend, but we hope you had a relaxing weekend wherever you are! This week, we're talking about medical treatment for your kids. What was your experience when your child was sick and needed treatment to help their breathing? What went well? What could have been better? What worried you? What was reassuring? Please tell us in the comments below!

- Medicine can help our kids a lot, but sometimes it comes along with annoying, or even scary, side effects. Has your child gone through this? Did it get bad enough to stop treatment, or were they okay to wait it out? Any lasting effects? A combination? Share your experience and how you felt in the comments below.

- What goes through your mind when your child is prescribed a treatment? Relief? Possible long-term effects? That it's the right one? A combination? Tell us your thoughts in the comments below!

<u>Week 4: February 22 – 26, 2016</u>

Theme: **Course of Illness**

Sometimes when children are sick, they are not themselves.

1. Tell us about symptoms you find the most concerning when your child is sick and their breathing is affected. *(Common symptoms are things like coughing, wheezing, fever, sore throat, headache, and struggling to breathe)* Why?
    a. What about things you're not too worried about? Why not?
2. How important to you is the amount of time that it takes your child to feel better?
    a. Is there a point in time when you're not concerned versus a point in time when you become concerned?

For peer review only - http://bmjopen.bmj.com/site/about/guidelines.xhtml

Supplementary File 1

3. How concerned would you be if your child got sick again with the same illness, after feeling better?

- Sometimes when children are sick, they are not themselves. Respiratory infections can mean some nasty symptoms for kids, like coughs, fevers, and sore throats. What symptoms do you find most concerning when your child is sick? Tell us in the comments below!

- Parents, how do your dinosaurs get well soon?! Tell us what your kids are like when they're sick in the comments below!

- Respiratory infections can bring anyone down for a while. How do your kids cope? #BringBackFridayFunDay

Week 5: February 29 – March 4, 2016

Theme: **Financial Burden**

Sometimes when children are sick, parents or caregivers need to pay for extra things.

1. How concerned are you about the costs that come up when your child is sick?
   a. Costs related to treatment, like medications, supplies, or doctor or nurse visits?
   b. Other costs that came up while your child was sick, like child care, parking, or lost income for missing work?

- Happy Monday, Moms and Dads! This week, we're talking about costs that might come up when your kids are sick. Tell us what has come up for you in the comments below!

- There's a lot to think about when your child is sick. How much do you think about extra costs? Maybe that looks like costs for treatment, or maybe it's something like lost income for missing work. Share your thoughts in the comments below!

- Those darn hidden costs! Tell us what gets to you in the comments below!

Calvin and Hobbes, by Bill Watterson

Week 6: March 7 – 11, 2016

Theme: **Daily Activities and Routines**

Sometimes when children are sick, we can't follow a regular schedule or do regular activities.

For peer review only - http://bmjopen.bmj.com/site/about/guidelines.xhtml

Supplementary File 1

1. Are there any regular activities that you'd be concerned about if your child couldn't do them when they're sick and their breathing is affected?
    a. How concerned would you be about your child:
        i. Not eating or drinking well?
        ii. Not getting as much sleep as they normally do?
        iii. Being away from school or daycare?
    b. How concerned would you be about:
        i. Having to take time off work?
        ii. Arranging child care for your sick child or other children?
2. Are there any other activities that you'd be concerned about disruptions to?

- Welcome to another week of the OUTCH Study! Thanks for checking us out. This week, we're wondering about how your regular routine is disrupted when your child is sick. What happens in your family that's out of the ordinary? Tell us below!

- Sound familiar? Are there activities that you and your little ones miss when they are sick?

Just a Little Sick, by Mercer Mayer

- Thanks for the great discussion this week! Sounds like sleep is at the top of a lot of parents' lists of routines that are disrupted when their child is sick - although "everything" would also probably cover it! What else happens in your family? Tell us in the comments below!

<u>Week 7: March 14 – 18, 2016</u>

Theme: **Complications**

It can sometimes be scary when our children have health complications because their breathing is affected

1. How concerned would you be if your child had mild complications like a cough or a rash?
    a. What was or would be most concerning about your child having mild complications?
2. How concerned would you be if your child had severe complications like needing to be connected to a ventilator to help them breathe?
    a. What was or would be most concerning about your child having severe complications when they were sick and their breathing was affected?

- Welcome back, Moms and Dads! Sometimes when children are sick, complications come up, which can be scary. Complications might be things like a cough or a rash, or could be more serious like needing to

For peer review only - http://bmjopen.bmj.com/site/about/guidelines.xhtml

Supplementary File 1

be connected to a ventilator. Have your children gone through this? Please share your experience below.

- This week, we're talking about complications. Here's one mom's story:

He had all the signs of a cold coming on. With the rest of his siblings having been sick over the last few weeks, it was no surprise. He was super cranky all day and by supper time, he wasn't eating or drinking much. He'd had colds before but he wasn't acting well and looked tired. It all happened so fast - a drive to the hospital and 5 hours later, he was hooked up to a machine to help him breathe. The doctor said he had RSV and would likely need to stay a few days to be monitored and recover. I can't forget the helplessness I felt, watching him fighting to breathe, needing to be connected to the ventilator.

What about you? Please share your story in the comments below.

- Hey Parents, has your child had any complications when they've been sick with a respiratory infection? Tell us about it in the comments below.

<u>Week 8: March 21 – 25, 2016</u>

Theme: **Closing**

1. Please tell us any other things that are important to you when your child is sick with an illness that affects their breathing that we have not talked about yet.

- Happy Monday, everyone, and welcome to the last week of the OUTCH Study! Until next Sunday, tell us what matters to you when your child is sick with a respiratory infection. Is it their symptoms? Needing to see a healthcare provider? Disruptions to normal routines? Share what hits home for you in the comments below!

- Hey Parents, help us make sure we're not missing anything! What is most important to you when your child has a respiratory infection? Anything that's on your mind – we want to know!

- Thanks, everyone, for taking part in the OUTCH Study! Your input means a lot to us as we continue to try to make child health research and care better!

For peer review only - http://bmjopen.bmj.com/site/about/guidelines.xhtml

Which outcomes are important to patients and families who have experienced pediatric acute respiratory illness? Findings from a mixed methods sequential exploratory study

Journal:	*BMJ Open*
Manuscript ID	bmjopen-2017-018199.R1
Article Type:	Research
Date Submitted by the Author:	16-Oct-2017
Complete List of Authors:	Dyson, Michele; University of Alberta, Department of Pediatrics<br>Shave, Kassi; University of Alberta, Department of Pediatrics<br>Gates, Allison; University of Alberta, Department of Pediatrics<br>Fernandes, Ricardo; Hospital de Santa Maria, Departamento da Criança e da Família; Faculdade de Medicina de Lisboa, Instituto de Medicina Molecular, Laboratorio de Farmacologia Clinica e Terapeutica<br>Scott, Shannon; University of Alberta, Nursing<br>Hartling, Lisa; University of Alberta, Pediatrics
**Primary Subject Heading**:	Paediatrics
Secondary Subject Heading:	Patient-centred medicine, Respiratory medicine, Infectious diseases
Keywords:	Community child health < PAEDIATRICS, Paediatric infectious disease & immunisation < PAEDIATRICS, RESPIRATORY MEDICINE (see Thoracic Medicine), Paediatric thoracic medicine < THORACIC MEDICINE, Respiratory infections < THORACIC MEDICINE

SCHOLARONE™ Manuscripts

For peer review only

For peer review only - http://bmjopen.bmj.com/site/about/guidelines.xhtml

Which outcomes are important to patients and families who have experienced pediatric acute respiratory illness? Findings from a mixed methods sequential exploratory study

Michele P Dyson, Kassi Shave, Allison Gates, Ricardo M Fernandes, Shannon D Scott, Lisa Hartling

Michele P Dyson, Assistant Professor, Department of Pediatrics, University of Alberta

Kassi Shave, Former Research Assistant, Alberta Research Centre for Health Evidence (ARCHE), Department of Pediatrics, University of Alberta

Allison Gates, Research Associate, Alberta Research Centre for Health Evidence (ARCHE), Department of Pediatrics, University of Alberta

Ricardo M Fernandes, Pediatrician, Department of Pediatrics, Hospital de Santa Maria, Lisbon

Shannon D Scott, Professor, Faculty of Nursing, University of Alberta

Lisa Hartling, Professor, Department of Pediatrics, University of Alberta

Correspondence to: Dr. Michele P Dyson, 4-474 Edmonton Clinic Health Academy, University of Alberta, 11405 – 87 Avenue, Edmonton, Alberta, Canada T6G 1C9, E-mail: mdyson@ualberta.ca, Phone: 1.780.492.1241

For peer review only

For peer review only - http://bmjopen.bmj.com/site/about/guidelines.xhtml

ABSTRACT

Objectives. To identify the outcome priorities of parents of children who had experienced an acute respiratory infection (ARI).

Design. This was a two-phase, mixed methods study with a sequential exploratory design. We used a cross-sectional quantitative web-based survey to elicit parents' priorities for pediatric ARI. We then used a discussion moderated via Facebook to elucidate richer descriptions of parents' priorities.

Setting. Survey and discussion data were collected via the Internet.

Participants. 110 parents (90% female, median age 35 years, 92.7% urban dwelling, 94.5% with a post-secondary education) with a child who had experienced an ARI responded to the survey. Four parents participated in the Facebook discussion.

Primary and secondary outcome measures. The primary outcome was parents' rankings of outcomes related to pediatric ARI. The secondary outcomes were the alignment of parent-reported important outcomes with those commonly reported in Cochrane systematic reviews (SRs).

Results. Commonly reported ARIs included croup (44.5%), wheezing (43.6%), and influenza (38.2%). Parents ranked major complications, illness symptoms, and length of stay as the most important outcome categories. With respect to specific outcomes, severe complications, major side effects, doctor's assessment, relapse, oxygen supplementation, and results from lab measures were reported as most important (75th-99th percentile). Taking time off work, mild complications, interference with daily activities, treatment costs, absenteeism, follow-up visits and other costs were deemed minimally important (<25th percentile). In 35 Cochrane SRs, 29 unique outcomes were reported. Although participants' priorities sometimes aligned with outcomes frequently reported in the literature, this was not always true. Additional priorities from the survey (n=50) and Facebook discussions (n=4) included healthcare access, interacting with healthcare providers, education, impact on daily activities, and child wellbeing.

Conclusions. In the context of pediatric ARI, parents' priorities did not always align with commonly researched outcomes. Appealing and efficient strategies to engage patients and parents in research should be developed.

Key Words: social media, child health, patient-centered outcomes research, stakeholder engagement

For peer review only - http://bmjopen.bmj.com/site/about/guidelines.xhtml

STRENGTHS AND LIMITATIONS OF THIS STUDY

- The identification of patient-important outcomes is a necessary precursor to the conduct of research that is relevant to themselves and their families, though engaging patients in research is challenging.
- We used web-based tools and social media platforms to recruit and engage patients and identify patient-important outcomes for pediatric acute respiratory infections.
- Though nearly three-quarters of online adults use social media, engaging in health research online may appeal only to certain sub-populations, so the findings may not be generalizable.

For peer review only

For peer review only - http://bmjopen.bmj.com/site/about/guidelines.xhtml

INTRODUCTION

The determination of outcomes that matter to patients is foundational to the conduct of research that is relevant to them and their families. With an increasing emphasis on patient-centeredness in clinical research, numerous organizations and strategies have been established with this as their mandate (e.g., Canada's Strategy for Patient-Oriented Research [1], the Patient-Centered Outcomes Research Institute in the United States [2], and INVOLVE in the United Kingdom [3]). Involving patients in the research process will ensure that funded investigations utilize questions, outcomes, and interventions that are aligned with their needs and priorities [4-6].

There are many complexities involved in selecting outcomes, and little published guidance for investigators exists [7]. There is significant heterogeneity in the outcomes measured and reported in studies of specific diseases, which may in part occur due to uncertainty around which outcomes are patient-important [7]. The development of core outcome sets, in which a minimum group of agreed-upon outcomes is measured and reported on across clinical research in a specific condition, has been proposed as a solution to these issues [8]. Sinha et al. [7] identified 13 groups formed to develop core outcome sets for pediatric clinical trials, including the Core Outcome Measures in Effectiveness Trials (COMET) and Outcome Measures in Rheumatology (OMERACT). The COMET initiative was launched in 2010 to bring together individuals interested in developing core outcome sets, and to collate outcome sets and relevant resources [8]. Established in 1992, OMERACT is a consensus initiative that has developed a number of widely utilized core outcome sets for rheumatologic conditions, with patients actively involved in the process since 2002 [9, 10].

One criticism of commonly used methods to develop core outcome sets is that they do not include a systematic survey of stakeholders [9]. Social media represents a medium where patients and their caregivers increasingly interact online [11, 12], providing an opportune channel for engagement in the development of core outcomes. Nearly three quarters (74%) of online adults use social media, with Facebook continuing to be the most popular social media site, and multi-platform use increasing in prevalence [13, 14]. Despite the global pervasiveness of social media, its use for engaging patients and/or caregivers in the outcome selection process has not extensively been explored.

We conducted a two-phase, sequential exploratory mixed methods study using social media to recruit and engage the parents of children with an acute respiratory infection (ARI) to elucidate patient-

For peer review only - http://bmjopen.bmj.com/site/about/guidelines.xhtml

important outcomes. Acute respiratory infections are common among children, represent a significant source of morbidity, and are one of the leading causes of illness, emergency department visits, and hospitalization [15, 16]. There is a scarcity of research on patient perspectives in this area [7]. Using social media, we aimed to: (a) recruit and survey parents to identify their priorities for ARI outcomes as compared to those commonly reported in the literature, and (b) engage parents in discussions to elucidate the rationale for their priorities, as identified in the quantitative survey.

METHODS

This mixed methods study utilized an explanatory sequential design [17], and involved two phases: 1) a quantitative survey to determine parent priorities for ARI outcomes; 2) a qualitative follow-up in which we sought elaboration on parents' priorities. A process evaluation of our social media strategy is reported elsewhere [18].

Institutional Ethics Approval

Prior to beginning the study, we sought and received ethical approval for both the quantitative and qualitative components from the University of Alberta Research Ethics Board (# Pro00058629). Parents and/or caregivers were eligible to participate if they had a child aged 0-17 years who had experienced one or more episodes of acute asthma, bronchiolitis, croup, influenza, strep throat/tonsillitis, pneumonia, sinusitis, and/or wheezing. Participant consent was implied through overt action by completing the survey or publishing public responses online. Prior to participation, we provided interested potential participants with an information letter that described the study and explained consent via overt action. Participants were free to withdraw, end, or modify their participation in the study at any time without consequence, and we retained any data collected only with their permission.

Phase I: Quantitative Survey

Development and Pre-Testing

The first draft of the survey was informed by previous research on the outcomes that are important to clinicians and families of children with asthma [19] and by outcomes frequently reported in the literature. To determine the frequency of outcomes reported in the literature, we identified systematic reviews (SRs) published up to 2013 from the Cochrane Database of Systematic Reviews (n=35) and their included studies. We grouped frequently reported outcomes and those previously identified as important [7, 19, 20] into seven broad categories: disease activity; disease complications; adverse

For peer review only - http://bmjopen.bmj.com/site/about/guidelines.xhtml

For peer review only

effects of therapy; functional status; social and family outcomes, including quality of life; long-term effects of interventions; and resource utilization. We sent a draft of our survey to a group of 10 clinicians/researchers and 8 parents who reviewed it independently and provided feedback on its content and comprehensibility. We analysed the written feedback qualitatively. Based on the content analysis, our research team revised the original categories, finally deciding on ten categories of outcomes for the survey. These included outcomes that were identified as important by clinicians and parents that were missing from the draft. The ten categories on the final version of the survey included: major complications; symptoms; length of stay in the emergency department or hospital; needing to see a doctor; returns visits to a doctor or the hospital; reactions to medications; medical test results; maintenance of day-to-day activities; minor complications; and cost of illness. The survey is available as a supplementary file (Supplementary File 1).

We ensured survey accessibility across different operating systems, including touch screen (e.g., tablets, smartphones) or keyboard (e.g., desktop computers) technology. We wrote the materials at a sixth grade reading level and conducted pilot testing with 8 parents and 10 researchers/clinicians to ensure that the language and flow of questions were appropriate.

Recruitment

We created an online and social media presence via a study website (www.outch-study.com), a Facebook page (OUTCH), and a Twitter account (@OUTCH_Study). We used snowball sampling [21] to recruit parents. First, we focused on identifying and engaging recruitment targets with the potential for a high yield of participants. We then expanded our scope through referrals and diffusion via social media. Tactica Interactive (http://tactica.ca), a digital media enterprise, was hired to broaden our sampling frame via a Facebook advertising strategy.

We collaborated with organizations interested in ARI and patient engagement to advertise our research via websites and other channels: The Alberta Centre for Child, Family & Community Research (ACCFCR, now known as PolicyWise for Children and Families; a provincial organization linking government, academia, and the community in a focus on evidence-informed policy and practice) [22], TRanslating Emergency Knowledge for Kids (TREKK; a national network of researchers and clinicians invested in improving pediatric emergency care) [23]; the Cochrane Consumer Network (CCNet; an international network of healthcare consumers with an interest in evidence-based medicine) [24]; and the Stollery

For peer review only

Family Centered Care Network (a local children's hospital-based network of patients and families that provide input into patient care) [25]. We also engaged an online (Facebook and Twitter) parenting community, Mommy Connections, that regularly promoted the study through their networks.

Data Collection

The quantitative survey was administered by Nooro Online Research (https://nooro.com/index.html) for 14 weeks from December 2013 to March 2014. Links to the survey were provided through the study website, Facebook, and Twitter accounts and were completed anonymously. The only identifying information was an optional e-mail address for entry into a prize draw.

The survey included a combination of open- and closed-ended questions to determine the relative importance of outcomes currently measured in trials and SRs of ARI in children. The outcome categories were presented and parents were asked to identify their top five priorities from the list. Then, individual outcomes were presented and parents were asked to indicate their importance using a digital sliding scale, conceptually similar to a visual analog scale. The two ends of the scale were anchored with the statements 'Not important [/concerning] at all' and 'Extremely important [/concerning].' Parents were also asked to indicate additional items that were considered important to patients and their families, but may not have been addressed in the literature. The survey platform was also used to collect demographic data.

Phase II: Qualitative Follow-Up

In the second phase, we conducted an Internet-based, descriptive qualitative study [26] to interact with parents and elicit elaboration on their perceptions of the importance of ARI outcomes. This study occurred across an eight-week period from January to March 2016 during which parents were engaged in discussion through an open online focus group hosted on the study Facebook page.

Recruitment

To recruit parents, we utilized a snowball sampling technique [21]. We first asked potentially high yield sources of participants to promote our study, including local and national online parenting communities (n=16), children's hospitals and associated foundations (n=14), and patient groups (n=3) with access to a large consumer audience. We also asked individuals and organizations within our existing networks to promote the study. Recruitment occurred throughout the study period, and was almost exclusively

For peer review only - http://bmjopen.bmj.com/site/about/guidelines.xhtml

carried out through Facebook. Some organizations or individuals promoted the study on Twitter or a blog, but all links posted drove user traffic back to the study Facebook page.

One variable of interest in this study was the reach of social media as a recruitment strategy (reported elsewhere [18]); therefore, the sample size was an outcome, rather than a pre-defined condition. To accommodate this, we did not define the number of participants *a priori*, instead allowing the detail that emerged from our data collection to guide the extent of recruitment. However, we did aim to recruit a sample size as guided by the principles of data saturation, in which data would be collected until no new themes emerged [27].

Data Collection

Throughout the study period, each week had a discussion theme modelled after the structure of the survey, and posts were published daily covering varying aspects of this theme. Three different types of posts were published: promotional posts prior to the study launch; parent-friendly content about ARIs; and discussion questions where parents were encouraged to share their thoughts and experiences. The online focus group was moderated by two members of the study team (MPD, KS) and all posts by participants were followed up promptly with a response. An interview guide (Supplementary File 2) was developed to guide weekly topics and for reference during discussion moderation.

Data Analysis

Quantitative Analysis

Survey data were analyzed using SPSS (v. 22.0, IBM Corporation, Armonk, NY) and described using descriptive statistics (mean±SD; rank order). To determine the rank order of the outcomes most important to parents, we allocated each outcome 10 points when it was chosen as a top concern, and 8, 6, 4, and 2 points if it was chosen as the second, third, fourth, or fifth most important concern, respectively. After tallying the points for each of the 10 outcomes, we ordered these from largest to smallest to develop the ranked priority list.

To compare the published literature to the patient-important outcomes identified by parents, we collated a list of all of the outcomes, and calculated the number of SRS in which each outcome had been reported. We then grouped all of the outcomes into percentile ranges based on the number of SRs in which each was reported (‘frequently reported’: 75th-99th percentile; ‘moderately reported’: 25th-74.9^{th}

For peer review only - http://bmjopen.bmj.com/site/about/guidelines.xhtml

percentile; and 'infrequently reported': <25th percentile). Parents' rankings of the importance of outcomes were grouped similarly ('most important': 75th-99th percentile; 'moderately important': 25th-74.9th percentile; and 'least important': <25th percentile), and all comparisons were strictly descriptive. Parents who had healthcare insurance were self-identified in the survey and excluded from all analyses related to healthcare costs as these may not hold relevance.

Qualitative Analysis

Content posted by participants during the focus groups was extracted verbatim to form transcripts. These, along with the open-ended responses to the survey, were imported into NVivo 10 (QSR International, Melbourne, Australia) data management software. Data were analyzed inductively for themes. Two investigators (MPD, KS) participated in coding, following a three stage process: 1) reading through the data, making notes on themes and significance that were then compiled into a preliminary version of the codebook; 2) rereading the data and coding using the concepts identified in the first phase; and 3) refining and applying the codes to the text upon a third review [28]. Coders met to discuss progress and reach consensus on differing interpretations. Data collection and analysis occurred concurrently, following an iterative process to monitor progress and allow for follow up on ideas as they emerged.

RESULTS

Demographic Characteristics

A total of 110 people responded to the survey (Table 1). The survey website received 5,207 visits, a view rate (i.e., the ratio of unique survey visitors/unique site visitors) of 4.1% (205/5,207), and a completion rate (i.e., the ratio of unique visitors who completed the survey/users who agreed to participate) of 53.7% (110/205). A detailed account of the traffic to the survey and usability are available in our published process evaluation [18].

Ninety percent (n=99) of respondents were female. The median age was 35 years, and 89% (n=97) of respondents had received a college/university or post-graduate education. Most survey respondents were married (n=98; 89%), urban-dwelling (n=102; 93%), and resided in Canada (n=77; 70%). More than half of respondents reported an annual household income of >$90,000 USD (n= 69; 63%). Respondents were predominantly parents (n=106; 96%), and had a median of two children in the home (range 0-4). The respondents' children most commonly experienced croup (n=49; 45%), wheezing (n=48; 44%), and

For peer review only - http://bmjopen.bmj.com/site/about/guidelines.xhtml

For peer review only

influenza (n=42; 38%). The most concerning ARIs were croup (n=20; 25%), pneumonia (n=16; 20%), and asthma (n=15; 19%). The median year that the ARI occurred was 2012 (range 1994-2013), when the child was 1-year-old (range <1 month-10 years old). Most children did not have a chronic illness (n=90; 82%) and did not experience a hospital admission due to this ARI (n=84; 80%).

Table 1. Survey participant demographics (N=110)

**Characteristic**	**N (%)**
Gender	Female: 99 (90.0)<br>Male: 11 (10.0)
Age (years) (median (range))	35 (18-67)
Highest level of education	Some high school: 1 (0.9)<br>High school graduate: 5 (4.6)<br>Some college/university: 7 (6.4)<br>College/university graduate: 50 (45.5)<br>Post-graduate education/degree: 47 (42.7)
Marital status	Single: 5 (4.6)<br>Married/Common-law: 98 (89.1)<br>Separated/divorced/widowed: 7 (6.4)
Annual household income (USD$)	<30,000: 5 (4.6)<br>30-49,999: 7 (6.4)<br>50-69,999: 15 (13.6)<br>70-89,999: 14 (12.7)<br>>90,000: 69 (62.7)
Country of residence	Australia: 2 (1.8)<br>Canada: 77 (70.0)<br>England: 8 (7.3)<br>India: 2 (1.8)<br>Portugal: 2 (1.8)<br>United States: 19 (17.3)
Type of community	Urban (≥10,000): 102 (92.7)<br>Rural (<10,000): 7(6.4)<br>Missing: 1 (0.9)
Number of children in home (median (range))	2 (0-4)
Relationship to child	Parent: 106 (96.4)<br>Step-parent: 0 (0)<br>Grandparent: 4 (3.6)<br>Other: 2 (1.8)
Type of ARI	Bronchiolitis: 29 (26.4)<br>Croup: 49 (44.6)<br>Strep throat/tonsillitis: 36 (32.7)<br>Wheezing: 48 (43.6)<br>Influenza: 42 (38.2)<br>Pneumonia: 24 (21.8)

For peer review only - http://bmjopen.bmj.com/site/about/guidelines.xhtml

Characteristic	N (%)
	Asthma: 29 (26.4)<br>Other: 23 (20.9)
Most concerning ARI	Bronchiolitis: 10 (12)<br>Croup: 20 (25)<br>Strep throat/tonsillitis: 3 (4)<br>Wheezing: 6 (7)<br>Influenza: 2 (3)<br>Pneumonia: 16 (20)<br>Asthma: 15 (19)<br>Other: 6 (7)
Year of ARI (median (range))	2012 (1994-2013)
Child age at time of ARI (median (range))	1 year (<1 month-10 years)
Hospital admissions due to ARI	Yes: 21 (19.1)<br>No: 84 (76.3)<br>Missing: 5 (4.5)
Chronic illness	Yes: 20 (18)<br>No: 90 (82)

ARI: Acute Respiratory Infection

Quantitative Outcome Rankings

The overall ranking of categorized outcomes is shown in Table 2. On average, parents ranked major complications from the child's illness (e.g., long-term disability), illness symptoms (e.g., coughing, fever, sore throat), and length of stay in the emergency department or hospital as the most important outcome categories. Of least importance were the costs of their child's illness (e.g., medicine or child care), minor complications from the child's illness (e.g., cough or rash), and maintenance of day-to-day activities. The overall ranking of individual outcomes revealed that parents were most concerned about severe complications (mean score on a scale from 1-100: 94.5), major side effects (86.7), and their doctor's assessment (83.9). Other costs (e.g., child care, parking, lost income) (31.3), scheduled follow-up visits (38.1), and school/daycare absenteeism (40.4) received the lowest mean scores. When parents were grouped according to ARI their child had experienced and their rankings of the importance of outcomes were compared, Spearman correlation coefficients revealed strong agreement, indicating that perceptions of importance were consistent across conditions (Table 3).

Table 2. Overall ranking of categorized items (N=110)

Rank Order[a]	Category
**1**	Major complications from child's illness (e.g., long-term disability)
**2**	Illness symptoms (e.g., coughing, fever, sore throat)
**3**	Length of stay in the emergency department or hospital

For peer review only - http://bmjopen.bmj.com/site/about/guidelines.xhtml
For peer review only

**4**	Child needing to see a doctor
**5**	Return visits to the doctor or hospital
**6**	Child's reaction to his or her medicine (e.g., side effects)
**7**	Child's medical test results
**8**	Maintenance of day-to-day activities
**9**	Minor complications from child's illness (e.g., cough or rash)
**10**	Costs of child's illness (e.g., medicine or child care)

[a]Ordered from most to least important to parents.

Table 3. Agreement between acute respiratory infection type and parent ranking of categorized outcomes (N=110)

**Type of Acute Respiratory Infection**	**Spearman Correlation Coefficient***
Bronchiolitis	0.94
Croup	0.75
Strep Throat/Tonsillitis	0.66
Sinusitis	0.87
Wheezing	0.76
Influenza	0.66
Pneumonia	0.85
Asthma	0.67
Other	0.50

*0-0.2: poor/slight agreement; 0.2-0.4: fair agreement; 0.4-0.6: moderate agreement; 0.6-0.8: substantial agreement; 0.8-1: near perfect agreement

A comparison of the outcomes reported in the literature to those reported as important by parents is shown in Table 4. We extracted 221 outcomes from the 35 Cochrane SRs; the same outcomes were often reported in more than one SR. Out of the full list of reported outcomes, we isolated 29 individual outcomes each of which was reported in 1 to 26 of the SRs. Adverse events were the most frequently measured outcome (f=26; 11.8%) in our sample of Cochrane SRs, and similarly, severe complications (score: 94.5/100) and major side effects (86.7) were ranked as the most important (75th-99th percentile) to parents. Likewise, parents ranked returning to school/work and the cost of treatment as least important (40.4 and 44, respectively; <25th percentile), and these outcomes were infrequently measured in Cochrane SRs (f=2; <25th percentile).

There were many discrepancies between the outcomes measured in Cochrane SRs and the outcomes parents ranked as important. Relapse and the need for oxygen supplementation were ranked among the most important outcomes by parents (81.8 and 81.6, respectively; 75th-99th percentile), but were

For peer review only - http://bmjopen.bmj.com/site/about/guidelines.xhtml

measured moderately frequently (f=8 and f=5, respectively; 25th-74.9th percentile) in Cochrane SRs. Similarly, the results from lab measures were ranked highly by parents (81.4; 75th-99th percentile), but were infrequently reported in Cochrane SRs (f=4; 25th-74.9th percentile). The need for medication was one of the most frequently reported outcomes in Cochrane reviews (f=13; 75th-99th percentile), but was ranked as moderately important by parents (55.1; 25th-74.9th percentile). Admission rate was the second most frequently measured outcome in Cochrane SRs (f=19; 75th-99th percentile), yet was also ranked less favorably among parents (76.9; 25th-74.9th percentile). While the doctor's assessment of how the child is doing was ranked as the third most important outcome (83.9; 75th-99th percentile) by parents, only clinical scores/symptom scores, one of three corresponding components of this outcome (clinical scores/symptom scores; patient improvement; observed response to treatment) was measured frequently (f=15; 75th-99th percentile) in Cochrane SRs. Patient improvement was measured moderately frequently (f=6; 25th-74.9th percentile), and observed response to treatment was measured the least frequently of any outcome included in this study (f=1; <25th percentile).

Table 4. Parent ranking of individual outcomes compared to frequency of measurement in Cochrane systematic reviews

Outcomes reported in Cochrane systematic reviews (N=35)	Frequency of reporting	Parent ranking of importance of outcomes (N=110)	Mean Score ±SD (/100)
**75th-99th percentile**			
Adverse events (local and systemic)	26	Severe complications	94.5 ±14.5
Admission rate (hospital, ED, ICU)	19	Major side effect	86.7 ±18.9
Physical signs	18	Doctor's assessment	83.9 ±19.6
Clinical measures	17	Relapse	81.8 ±18.4
Clinical scores/symptom scores	15	Oxygen supplementation	81.6 ±21.6
Length of stay/time to discharge (hospital, ED, ICU)	15	Results from lab measures	81.4 ±19.4
Need for medication	13		
**25th-74.9th percentile**			
Severity of symptoms	12	Length of hospital stay	78.6 ±22.1
Duration of symptoms	10	Trip to emergency department	77.6 ±23.9
Complications	9	Time to recovery	77.4 ±17.8
Rates of relapse	8	Hospital admission	76.9 ±23.7
Clinical treatment failure	7	Return healthcare visit	76.9 ±24.0
Patient improvement	6	Not eating/drinking well	65.9 ±22.8
Time to resolution of illness/time to recovery	6	Lack of sleep	63.2 ±20.0
Duration of oxygen supplementation	5	Length of stay in emergency department	62.4 ±26.1
Mortality	5	Minor side effect	55.1 ±24.8
Laboratory measures	4	Prescription for medication	55.1 ±25.6
Readmission	4	Appointment with	48.4 ±27.8

For peer review only

For peer review only - http://bmjopen.bmj.com/site/about/guidelines.xhtml

		GP/pediatrician	
		Arranging child care	48.3 ±30.1
**<25th percentile**			
Clinical cure	3	Taking time off work	47.2 ±28.4
Compliance and tolerance	3	Mild complications	46.3 ±23.3
Quality of life/patients' well-being	3	Interference with daily activities	44.6 ±23.5
Return healthcare visits	3	Treatment costs	44.0 ±32.5
Return to school/work	2	School/daycare absenteeism	40.4 ±26.5
GP visits	2	Scheduled follow-up visits	38.1 ±24.8
Treatment cost	2	Other costs	31.3 ±31.3
Adverse events that necessitated discontinuation of treatment	1		
Sleep disturbance	1		
Parental perception of child's status	1		
Observed response to treatment	1		

Qualitative Synthesis

A total of 50 respondents provided qualitative responses on the survey. Four participants contributed to the discussion on the Facebook page. The thematic analysis revealed five main analytical themes relating to parents' priorities and concerns when their child had an ARI: 'accessing health care'; 'interacting with healthcare providers'; 'illness education'; 'impact of illness on daily activities'; and 'child wellbeing'.

Accessing Health Care

Though not expressed by all parents, having timely access to health care for their child was a primary concern for many. Being able to get the medical advice they needed, without encountering substantial waiting times was important. Parents expressed concern about recognizing the signs and symptoms of their child's disease process. Determining whether their child's symptoms were serious or minor was considered challenging. Parents lacked confidence in deciding when to seek medical attention, particularly when the child frequently experienced breathing problems. Overall, parents shared wanting to avoid making unnecessary health care visits, and expressed the importance of receiving helpful tips from health care providers for managing acute symptoms at home.

Interacting with Health Care Providers

When parents accessed health care for their child, they desired to be taken seriously by healthcare providers. Parents described concern about their child's healthcare provider being dismissive or uninterested in their chief complaints about their child's health. Strong communication with healthcare providers was widely valued by parents. Parents expressed wanting to feel heard, and to have a medical

For peer review only

For peer review only - http://bmjopen.bmj.com/site/about/guidelines.xhtml

team that was both helpful and thorough in explaining their findings in a way that was direct and understandable.

Illness Education

Receiving education about their child's illness was widely regarded as important. Parents described requiring a complete understanding of what to expect, when to seek medical attention, and what the recovery time and process would be like. Information about the long-term impact of their child's health condition, and what effect recurrences or exacerbations might have, was regarded as of specific importance. When appropriate, parents regarded being presented with a variety of treatment options as critical. Parents expressed wanting to be involved in their child's recovery, and regarded education about their child's illness as essential to taking an active role.

Impact of Illness on Daily Activities

Parents described concern around interruption of their work schedules and sleeping routines. Being able to stay at home or in hospital with their child when they were unwell was important to parents, while not always possible when balancing financial and care responsibilities for other siblings. Disruption of sleep routines for parents and siblings was also described as concerning, particularly among parents of children with uncontrolled cough. Potential spread of their child's ARI between siblings and to the parents themselves was noted as a primary concern, and highly disruptive to daily activities.

Child Wellbeing

Parents shared their concern for their child's psychological wellbeing when they had an ARI. Concern about how their child was coping when they were unwell, particularly for those children with recurring or chronic ARI, was emphasized. Parents also expressed concern about how others treated their child when they were sick. One parent described concern over their child being treated like an 'invalid' when experiencing an acute asthma exacerbation.

DISCUSSION

Knowledge of patients' and their families' priorities is essential to guide the conduct of research that is relevant to themselves as well as to clinicians and policymakers [19]. Using social media, we engaged >100 parents over 14 weeks in a survey to elucidate the outcomes that they deemed most important with regard to pediatric ARIs. Parents' most important concerns included clinical outcomes like major

For peer review only - http://bmjopen.bmj.com/site/about/guidelines.xhtml

complications, symptoms, and length of stay in the emergency department or hospital. Psychosocial outcomes, and the ability of the family to cope during a child's illness, were also important. Not surprisingly, parents who participated in the focus groups were also concerned about process measures, like wait times, communication with healthcare providers, and managing their child's care at home. Although parents did not explicitly make the link, research has indicated that certain care processes, e.g., patient-centeredness, may contribute to better health outcomes [29]. For example, family centered care is associated with improved clinical outcomes for children and greater satisfaction with care [30]. The approach is characterized by honest communication between families and healthcare providers; policies and procedures that are tailored to the needs of families and children; ensuring support for families and children; and empowering them to participate in care decisions [30].

The maintenance of day-to-day activities was of relatively low importance to parents, seemingly contradicting the highly ranked importance of major complications and long-term disability. A previous study of asthma outcomes found that parents were more concerned about the long-term compared to short-term beneficial and harmful effects of therapy [19]. Because we did not quantify the temporality of the outcomes on the survey or in the discussions, we can only presume that parents interpreted the maintenance of day-to-day activities as a short-term outcome resulting from relatively minor illness. Although major complications can result in long-term or permanent changes to daily routines, they could also be potentially life threatening. When presented with the possibility of serious complications that could limit long-term quality of life, the maintenance of one's daily routine may seem relatively unimportant.

Health, though difficult to define, encompasses not only an individual's physical condition, but also their emotional, and psychological wellbeing [31]. Our survey of 35 Cochrane SRs revealed that a diverse array of health outcomes are being measured and reported, many of which are not aligned with those that are important to parents. These findings reinforce the growing recognition that insufficient consideration is being paid to the selection of outcomes within clinical trials [32]. Specifically, the need for core outcome sets [33], and especially ones that incorporate patient-reported and patient-centered outcomes [34] has garnered increased attention in recent years. More consistent reporting of outcomes for pediatric ARI will be necessary to facilitate evidence synthesis [32, 34], to enhance trustworthiness by reducing the risk of reporting bias [35], and to reduce research waste [36].

For peer review only - http://bmjopen.bmj.com/site/about/guidelines.xhtml

Good practice in clinical trials includes selecting a primary outcome that measures a clinically relevant and important treatment benefit [37]. Likewise, we found that the bulk of the research in child health focused on biological outcomes, with relatively little attention being paid the psychosocial impact of illness [20, 38, 39]. Still, there remains room in research for the measurement of outcomes important to patients and their families. Standards for Research in (StaR) Child Health, founded in 2009, brings together clinical and methodological experts to develop and promote the uptake of evidence-based guidance for child health research [40]. They assert that trialists should measure the effects of interventions more comprehensively; by measuring long-term outcomes and those that are relevant to decision-makers and families, the findings of trials will be of greater value [20].

Moving toward greater inclusion of patient-important outcomes in pediatric health research is challenged by the fact that children and their parents can be difficult to reach and engage. Given the pervasiveness of social media use via multiple platforms by patients and their caregivers [41], we postulated that this would provide an opportune medium to learn parents' perspectives. Though we experienced relative success in recruiting parents to complete the survey, qualitative engagement via the Facebook discussion group was more arduous. Moreover, despite moderate success in engaging parents, we did not gather any information from children themselves. Children have the right to participate in matters that affect their own lives [42], and can provide unique perspectives that cannot be elicited from their caregivers. Nevertheless, children also require protection, and the extent to which minors can understand and express their own healthcare needs remains controversial [42].

The challenges that we experienced are not unique. A review of studies that addressed the process of outcome selection identified only three studies that involved parents and none that involved children in the identification of pediatric patient-important outcomes [7]. In deciding which outcomes should be measured in pediatric ARI, it will be essential that stakeholders with varied perspectives, including parents, children, researchers, clinicians, and decision-makers convene and reach agreement on research priorities [32]. Suggested approaches like the Delphi technique and nominal group technique provide a means for stakeholders to reach unanimity on important outcomes in child health research [7,32]. These are, however, time consuming, resource-intensive and are highly burdensome to participants, which may limit recruitment and engagement. Further guidance is required on consensus methods that are efficient and appealing to patients, families and other stakeholders. Methods that are understandable to children will need to be developed if researchers are to uphold the rights of minors to

For peer review only

For peer review only - http://bmjopen.bmj.com/site/about/guidelines.xhtml

be involved in their own healthcare [42]. Reconciling children's and parents' perspectives, and the extent to which minors should be involved in the consensus process, requires further study.

Limitations

Our sample of parents and guardians were highly educated, many of whom had family incomes well above the national median, were mostly urban dwelling, and mainly Canadian, limiting the generalizability of the findings. For example, participants with lower incomes, or those residing in countries without publicly funded health care may have placed more importance on the cost of illness. As we did not provide any details to parents as to the temporality of the outcomes on our survey and in our discussions, we were not able to determine whether short- or long-term complications were more important to parents.

We had great difficulty engaging parents in the qualitative discussion and only elucidated responses from four participants. This seriously limited our ability to make informed inferences with regard to parents' quantitative ranking of the outcomes, leaving these mainly open to interpretation. For this reason, our understanding of the reasoning behind parents' ranking of the outcomes, and the content of the emergent qualitative themes are preliminary. Further work is required to develop a more comprehensive understanding of why some ARI-related outcomes are more important to parents than others.

CONCLUSIONS

The conduct and reporting of research of little relevance to the primary stakeholders represents a significant source of research waste, and appears prevalent in the context of pediatric ARI. The development of core outcome sets that include patient-important outcomes will facilitate evidence synthesis and reduce reporting bias, supporting the utility and trustworthiness of research findings. Future investigations are required to elucidate ways to make engagement in research more efficient and appealing to patients and their families.

ACKNOWLEDGEMENTS

The authors would like to thank Sarah Oberik-Olivieri for her graphic design work for this study.

COMPETING INTERESTS

For peer review only - http://bmjopen.bmj.com/site/about/guidelines.xhtml

All authors have completed the ICMJE uniform disclosure form at www.icmje.org/coi_disclosure.pdf and declare: no support from any organisations for the submitted work; no financial relationships with any organisations that might have an interest in the submitted work in the previous three years; no other relationships or activities that could appear to have influenced the submitted work.

FUNDING

This work was supported by KT Canada, grant number CIHR 87776 (SG-1) and Alberta Innovates Health Solutions, grant number 201400561. LH is supported in part by a Canadian Institutes of Health Research New Investigator Award. SS is a Canada Research Chair (Tier II) for Knowledge Translation in Child Health and is also supported by an Alberta Innovates Health Solutions Population Health Investigator Award. The funders had no role in study design, data collection and analysis, decision to publish, or preparation of the manuscript.

AUTHOR CONTRIBUTIONS

MPD and LH designed the study. MPD oversaw all aspects of the study's implementation. MPD and KS collected, analysed and interpreted the data with input from AG, LH, RMF, and SDS. All authors had full access to the data, and can take responsibility for the integrity of the data and the accuracy of the data analysis. KS and AG drafted the manuscript. MPD, LH, RMF and SDS reviewed the manuscript critically for intellectual content. All authors approved the version of the manuscript that was submitted to the journal. MPD affirms that the manuscript is an honest, accurate, and transparent account of the study being reported; that no important aspects of the study have been omitted; and that any discrepancies from the study as planned have been explained.

DATA SHARING STATEMENT

Anonymized data are available from the corresponding author upon reasonable request.

REFERENCES

For peer review only

1. Canadian Institutes of Health Research [Internet]. Strategy for Patient-Oriented Research. 2016 [cited 2017 May 1]. Available from: http://www.cihr-irsc.gc.ca/e/41204.html.
2. Patient-Centered Outcomes Research Institute [Internet]. 2016 [cited 2017 May 1]. Available from: http://www.pcori.org/.
3. National Institute for Health Research [Internet]. INVOLVE. 2015 [cited 2017 May 1]. Available from: www.invo.org.uk.
4. Frank L, Basch E, Selby JV. Patient-Centered Outcomes Research Institute. The PCORI perspective on patient-centered outcomes research. *JAMA* 2014;312(15):1513-4.
5. National Institute for Health Research [Internet]. INVOLVE. Exploring the impact of public involvement on the quality of research: examples. 2013 [cited 2017 May 1]. Available from: http://www.invo.org.uk/wp-content/uploads/2013/08/invoNETexamples2013.pdf.
6. Methodology Committee of the Patient-Centered Outcomes Research Institute (PCORI). Methodological standards and patient-centeredness in comparative effectiveness research: the PCORI perspective. *JAMA* 2012;307(15):1636-40.
7. Sinha I, Jones L, Smyth RL, et al. A systematic review of studies that aim to determine which outcomes to measure in clinical trials in children. *PLoS Med* 2008;5(4):e96.
8. COMET Initiative [Internet]. 2017 [cited 2017 May 1]. Available from: http://www.comet-initiative.org/.
9. Boers M, Kirwan JR, Wells G, et al. Developing core outcome measurement sets for clinical trials: OMERACT filter 2.0. *J Clin Epidemiol* 2014;67(7):745-53.
10. Boers M, Brooks P, Strand CV, et al. The OMERACT filter for Outcome Measures in Rheumatology. *J Rheumatol* 1998;25(2):198-9.
11. Fernandez-Luque L, Bau T. Health and social media: perfect storm of information. *Healthc Inform Res* 2015;21(2):67-73.
12. Fox S, Duggan M. Health Online 2013 [Internet]. 2013 [cited 2017 May 1]. Available from: http://www.pewinternet.org/files/old-media/Files/Reports/PIP_HealthOnline.pdf.
13. Duggan M, Ellison NB, Lampe C, Lenhart A, Madden M. Social Media Update 2014 [Internet]. 2015 [cited 2017 May 1]. Available from: http://www.pewinternet.org/2015/01/09/social-media-update-2014/.
14. Facebook [Internet]. Stats. 2017. [cited 2017 May 1]. Available from: http://newsroom.fb.com/company-info/.

For peer review only - http://bmjopen.bmj.com/site/about/guidelines.xhtml

15. Doan Q, Enarson P, Kissoon N, et al. Rapid viral diagnosis for acute febrile respiratory illness in children in the Emergency Department. *Cochrane Database Syst Rev* 2014;(9):CD006452.

16. Silka PA, Geiderman JM, Goldberg JB, et al. Demand on ED resources during periods of widespread influenza activity. *Am J Emerg Med* 2003;21(7):534-9.

17. Creswell JW, Plano Clark VL. Designing and conducting mixed methods research. Thousand Oaks, CA: Sage Publications; 2007.

18. Dyson MP, Shave K, Fernandes RM, et al. Outcomes in child health: Exploring the use of social media to engage parents in patient-centered outcomes research. *J Med Internet Res* 2017;19(3):e78.

19. Sinha IP, Gallagher R, Williamson PR, et al. Development of a core outcome set for clinical trials in childhood asthma: a survey of clinicians, parents, and young people. *Trials* 2012;13:103.

20. Sinha IP, Altman DG, Beresford MW, et al. Standard 5: selection, measurement, and reporting of outcomes in clinical trials in children. *Pediatrics* 2012;129 Suppl 3:S146-52.

21. Miles MB, Huberman AM. Qualitative data analysis: An expanded sourcebook. Thousand Oaks, CA: Sage Publications;1994.

22. PolicyWise for Children and Families [Internet]. About Us. 2017 [cited 2017 May 1]. Available from: https://policywise.com/about-us/.

23. TRanslating Emergency Knowledge for Kids (TREKK) [Internet]. About Us. 2017 [cited 2017 May 1]. Available from: http://trekk.ca/about.

24. Cochrane. Cochrane Consumer Network. 2017 [cited 2017 May 1]. Available from: http://consumers.cochrane.org/.

25. Alberta Health Services [Internet]. Family Centred Care (FCC). Stollery Children's Hospital. 2017 [cited 2017 May 1]. Available from: http://www.albertahealthservices.ca/services/page10093.aspx.

26. Sandelowski M. What's in a name? Qualitative description revisited. *Res Nurs Health* 2010;33(1):77-84.

27. Guest G, Bunce A, Johnson L. How many interviews are enough? An experiment with data saturation and variability. *Field Methods* 2006;18(1):59-82.

28. Guest G, MacQueen KM, Namey EE. Applied thematic analysis. Thousand Oaks, CA: Sage Publications; 2012.

29. Rathert C, Wyrwich MD, Austin Boren S. Patient-centered care and outcomes. A systematic review of the literature. *Med Care Res Rev* 2013;70:351-79.

For peer review only - http://bmjopen.bmj.com/site/about/guidelines.xhtml

30. Committee on Hospital Care; Institute for Patient- and Family-Centered Care. Patient- and family-centered care and the pediatrician's role. *Pediatrics* 2012;129:394-404.

31. Huber M, Knottnerus JA, Green L, et al. How should we define health? *BMJ* 2011;343:d4163.

32. Williamson PR, Altman DG, Blazeby JM, et al. Developing core outcome sets for clinical trials: issues to consider. *Trials* 2012;13:132.

33. Gargon E, Williamson PR, Altman DG, et al. The COMET initiative database: progress and activities update (2014). *Trials* 2015;16:515.

34. Macefield RC, Jacobs M, Korfage IJ, et al. Developing core outcomes sets: methods for identifying and including patient-reported outcomes (PROs). *Trials* 2014;15:49.

35. Dwan K, Gamble C, Williamson PR, et al. Systematic review of the empirical evidence of study publication bias and outcome reporting bias - an updated review. *PLoS One* 2013;8(7):e66844.

36. Chalmers I, Bracken MB, Cjulbegovic B, et al. How to increase value and reduce waste when research priorities are set. *Lancet* 2014;383(9912):156-65.

37. International Conference on Harmonisation of Technical Requirements for Registration of Pharmaceuticals for Human Use (ICH). Guidance for Industry. E9 Statistical Principles for Clinical Trials. Rockville, MD: US Department of Health and Human Services; Food and Drug Administration; Center for Drug Evaluation and Research; Center for Biologics Evaluation and Research; 1998.

38. Clarke SA, Eiser C. The measurement of health-related quality of life (QOL) in paediatric clinical trials: a systematic review. *Health Qual Life Outcomes* 2004;2:66.

39. Sinha IP, Williamson PR, Smyth RL. Outcomes in clinical trials of inhaled corticosteroids for children with asthma are narrowly focussed on short term disease activity. *PLoS One* 2009;4(7):e6276.

40. Van't Hoff W, Offringa M, Star Child Health group. StaR Child Health: developing evidence-based guidance for the design, conduct and reporting of paediatric trials. *Arch Dis Child* 2015;100:189-92.

41. Hamm MP, Chisholm A, Shulhan J, et al. Social media use among patients and caregivers: a scoping review. *BMJ Open* 2013;3(5):e002819.

42. Söderbäck M, Coyne I, Harder M. The importance of including both a child perspective and the child's perspective within health care settings to provide truly child-centred care. *J Child Health Care* 2011;15(2):99-106.

For peer review only - http://bmjopen.bmj.com/site/about/guidelines.xhtml

Supplementary File 1

1. **We are interested in learning which things you would be most concerned about if your child was sick with an illness that affected their breathing. By moving each item from the left side of the screen to the right side of the screen, please choose the top five things that would be most concerning to you. In your list, put the thing that would be most concerning to you at the top, followed by the next most concerning thing, and so on.** *(randomize order)*

__ If your child needed to see a doctor
__ How long your child needed to stay in the emergency department or hospital
__ If your child needed to return to the doctor or hospital after being sent home
__ Your child's test results that tell the doctor how he or she is doing
__ Your child's reaction to his or her medicine (for example, if it caused side effects)
__ Your child's symptoms (for example, coughing, fever, sore throat)
__ The costs of your child's illness (for example, for medicine or child care)
__ How well you and your child could keep up the day-to-day activities of your normal life
__ That your child could develop minor complications from their illness, such as a cough or rash
__ That your child could have major complications from their illness, such as a long-term disability

---

We would like to further explore the things that matter to you, as a parent, when your child is sick with an illness that affects their breathing. For the questions below, use the sliding scale to show how concerned you would be in each of the situations described.

Not at all concerned--*Very concerned* __ *Can't answer*
(this would not be an important concern for you if your child was sick) (this would be a very important concern for you if your child was sick)

<u>Getting Medical Help</u>

Sometimes when children are sick and their breathing is affected, they need help from a doctor. How concerned would you be about the following: *(Image 1)*

1. That you had to take your child to your family doctor or pediatrician's office.
2. That you had to take your child to the emergency department.
3. How long you had to stay at the emergency department.
4. That your child had to stay in the hospital (overnight or longer).
5. How long your child had to stay in the hospital.
6. That your child needed a scheduled follow-up visit, either with your family doctor/pediatrician or at the hospital.
7. That you had to go back see a doctor after the first time because he or she was not getting better. This could be at a doctor's office, a clinic, or the hospital.

<u>Tests and Treatment</u>

Sometimes children need tests when they are sick. How important are the following things to you: *(sliding scale: Not at all important – Very important) (Image 4)*

For peer review only

Supplementary File 1

8. Assessment of things like how fast your child was breathing, how fast his or her heart was beating, or whether he or she had a fever.
9. Results from x-rays, blood tests, or other tests, showing how well your child's lungs were working or what might be causing their illness.
10. The doctor's assessment of how your child was doing.

Sometimes when children are sick, they need to take medicine or be treated in other ways. How concerned would you be if: *(Image 5)*

11. Your child needed to be given extra oxygen through a mask or through his or her nose.
12. Your child needed to take medicine to feel better.
13. Your child had a minor but common side effect from the treatment or medicine. Some common side effects are vomiting, rash, and diarrhea.
14. Your child had a major side effect from the treatment or medicine that was bad enough to make him or her stop taking it. Some such reactions are your child becoming very agitated or irritable, or having a serious allergic reaction.

<u>**Other Concerns**</u>

Sometimes when children are sick, they are not themselves. How concerned would you be about the following things if your child was sick? *(Image 6)*

15. How bad your child's symptoms were. Symptoms are signs of your child's sickness. Common symptoms are things like coughing, wheezing, fever, sore throat, headache, and struggling to breathe.
16. How long it took for your child to feel better.
17. If your child got sick again with the same illness, after feeling better.

Sometimes when children are sick, parents or caregivers need to pay for extra things. How concerned would you be about: *(Image 7)*

18. How much your child's treatment cost, including costs for things related to treating their illness like medications, supplies, or doctor or nurse visits.
19. Other costs that came up while your child was sick, for example, child care, parking, or lost income for missing work.

Sometimes when children are sick, parents or caregivers can't follow their regular schedule or do regular activities. How concerned would you be about: *(Image 8)*

20. Your child not eating or drinking well because they felt sick.
21. You or your child not getting as much sleep as you normally do.
22. Your child being away from school or daycare.
23. The illness affecting the activities your child, you, or your family would normally do on a day-to-day basis.
24. Having to take time off of work.
25. Arranging child care either for your child who was sick, or arranging child care for your other children so that you could look after your sick child or take him or her to appointments.

For peer review only

Supplementary File 1

It can sometimes be scary when our children are sick. *(Image 9)*

26. How concerned would you be if your child had mild complications such as a cough or a rash?
27. How concerned would you be if your child had severe complications? This could mean something like your child being connected to a ventilator to help his or her breathing.

---

28. **Please tell us any other things that are important to you when your child is sick with an illness that affects their breathing that have not been included in this list.**

Please tell us a little bit more about your family.

29. **What is your gender?**
    __ Male
    __ Female
    (__ NR)

30. **What is your relationship to the child(ren) in your family? Check all that apply.**
    __ Parent
    __ Step-parent
    __ Grandparent
    __ Other, specify: __________
    (__ NR)

31. **How many children live in your home?** ___ (__ NR)

32. **What illness or illnesses that affect breathing has your child or children ever had? Please check all that apply.**
    __ Bronchiolitis
    __ Croup
    __ Strep throat (pharyngitis) or tonsillitis
    __ Sinus infection (sinusitis)
    __ Wheezing
    __ Flu (influenza)
    __ Pneumonia
    __ Acute asthma
    __ Other, specify: ______________
    __ I don't remember the name of the illness
    (__ NR)

    33a. If you checked more than one illness above, which one did your child have **most recently**?
    (drop down menu – same list as above) (__ NR)

For peer review only

Supplementary File 1

The following three questions will appear to correspond to conditions above

33b. **How old was your child when they had this illness?** *(drop down menu - <1/1/…/10/11 months/1/2/…18 years/can't remember)* (__ NR)

33b. When did this illness occur? MM/YYYY (__ NR)

33c. **Was your child admitted to the hospital, to stay overnight or longer?**
- __ Yes
- __ No
- (__ NR)

33d. **Does your child have any health concerns?**
- __ Yes, details: ________________
- __ No
- (__ NR)

33. **What year were you born in?** 19____ (__ NR)

34. **What is the highest level of schooling you have completed?**
- __ Grades 1-9
- __ Grades 10-11/Some high school
- __ High school graduate
- __ Some college/university
- __ College/university graduate
- __ Post-graduate education or degree
- (__ NR)

35. **What is your marital status?**
- __ Never married (single)
- __ Married/Common-law
- __ Separated, divorced, or widowed
- __ Other, specify: _________
- (__ NR)

36. **What is your household income (US dollars) per year?** (http://www.xe.com/currencyconverter/)
- __ Less than $30,000 per year
- __ $30,000-49,999
- __ $50,000-69,999
- __ $70,000-89,999
- __ Over $90,000
- (__ NR)

37. **What was your place of birth (town/city, country)?** _________ ________ (__ NR)

38. **Where do you live now (town/city, country)?** _______ ________ (__ NR)

For peer review only

Supplementary File 1

39. **How did you hear about this survey?**

__ Facebook
__ Twitter
__ Internet search (for example, Google)
__ Consumer or patient group (for example, Cochrane Consumer Network)
__ From a friend
__ From a health care professional (for example, a doctor or nurse)
__ Research network (for example, TREKK [Translating Emergency Knowledge for Kids])
__ Other, specify: ____________________
(__ NR)

For peer review only

Thank you for taking the time to fill out this survey!

If you would like to be entered into a draw for an iPad mini, a Kobo Touch, or a Nike+ FuelBand, click here to provide us with your email address. Your email address will not be linked to the responses you have provided.

Supplementary File 2

OUTCH Discussion Guide

Important Considerations

1. Is there agreement on the outcomes chosen, both among parents involved in the discussion, and between the discussion and the survey?
2. Are there areas (i.e., in current research) that have been overlooked?
3. What is the rationale for outcome selection?
4. What are considered to be clinically important or meaningful differences in the outcomes selected?

Week 1: February 1 – 5, 2016

Theme: **Getting Medical Help**

Sometimes when children are sick and their breathing is affected, they need help from a doctor.

1. Tell us about a time you needed to bring your child to a doctor because their breathing was affected.
    a. Did you have to go to see a doctor? Go to the emergency department?
    b. How did you feel?
    c. Did, or would, one have more impact on you than the other?
2. Tell us about what you find most concerning about getting medical help for your child when they are sick and their breathing is affected.
    a. Possible considerations (as prompts):
        i. how long you had to stay at the emergency department;
        ii. how long you had to stay in the hospital;
        iii. if you had to go for a scheduled follow-up visit to your family doctor or pediatrician;
        iv. if you had to go back to see a doctor after the first time because your child wasn't getting better.

- Thanks for checking out the OUTCH Study! We want to make sure that your child's health care is based on what's most important to you when their breathing is affected. This week, tell us what matters when your child needs to see the doctor. [attach graphic]

- Hey Parents! Have you had to take your child to the doctor or emergency department because they were having a hard time breathing? What was important to you?

- Excited to get this discussion going about what is important to you when your child is having a hard time breathing! Check out our page and share your experiences in the comments section.

Week 2: February 8 – 12, 2016

Theme: **Medical Tests**

Sometimes children need tests and treatment when they are sick.

For peer review only - http://bmjopen.bmj.com/site/about/guidelines.xhtml

Supplementary File 2

1. Tell us about a time your child needed tests because they were sick and their breathing was affected.
   a. What kind of tests did they need?
   b. At the time, how much did the results of things like x-rays or blood tests matter to you?
   c. How much did the doctor's assessment of your child's illness matter to you?
   d. Did, and why did, one type of assessment matter more to you than the other?

- Welcome to another week of the OUTCH Study! Help us understand your story. Tell us in the comments below about what matters to you when your child needs medical tests because they are having a hard time breathing. [attach graphic]

- If your child has needed tests like x-rays or blood tests when they're sick, what has mattered to you? Tell us in the comments below!

- Get to Know OUTCH!
We're asking you to share your stories with us, so we thought we'd tell you a little more about our team!

What does OUTCH stand for?
OUTCH stands for Outcomes in Child Health.

Who are we?
We're a group of researchers from the Department of Pediatrics at the University of Alberta, and we're the ones behind the scenes at the OUTCH Study.

What is the OUTCH Study all about?
We're interested in learning about outcomes that matter to you when your children's breathing is affected. This could mean that they have, or had, an illness like bronchiolitis, croup, strep throat or tonsillitis, a sinus infection, wheezing, the flu, pneumonia, or acute asthma. We want to talk to parents like you to make sure that our research asks the right questions, so we can improve healthcare for children and their families.

How can you help?
Tell us what matters to you in the comments section of our Facebook posts – we'd love to hear from you!

<u>Week 3: February 16 – 19, 2016 (*Family Day on February 15)</u>

Theme: **Medical Treatment**

Sometimes when children are sick, they need to take medicine or be treated in other ways.

For peer review only - http://bmjopen.bmj.com/site/about/guidelines.xhtml

Supplementary File 2

1. Tell us about a time when your child was sick and they needed treatment because their breathing was affected.
    a. What kind of treatment did they need?
        i. Were they given medicine? Oxygen?
2. How concerned were you about your child needing treatment?
    a. What was most concerning about your child needing treatment because their breathing was affected?
3. If your child has had more than one kind of treatment, have you found certain treatments more concerning than others?
4. How concerned would you be concerned about a mild side effect from the treatment or medicine, like vomiting, a rash, or diarrhea?
5. How concerned would you be about a major side effect from the treatment or medicine (like being very agitated or having a serious allergic reaction), that was bad enough that your child had to stop taking it?

- Welcome back parents! Here in Alberta we're just coming off of our Family Day long weekend, but we hope you had a relaxing weekend wherever you are! This week, we're talking about medical treatment for your kids. What was your experience when your child was sick and needed treatment to help their breathing? What went well? What could have been better? What worried you? What was reassuring? Please tell us in the comments below!

- Medicine can help our kids a lot, but sometimes it comes along with annoying, or even scary, side effects. Has your child gone through this? Did it get bad enough to stop treatment, or were they okay to wait it out? Any lasting effects? A combination? Share your experience and how you felt in the comments below.

- What goes through your mind when your child is prescribed a treatment? Relief? Possible long-term effects? That it's the right one? A combination? Tell us your thoughts in the comments below!

Week 4: February 22 – 26, 2016

Theme: **Course of Illness**

Sometimes when children are sick, they are not themselves.

1. Tell us about symptoms you find the most concerning when your child is sick and their breathing is affected. *(Common symptoms are things like coughing, wheezing, fever, sore throat, headache, and struggling to breathe)* Why?
    a. What about things you're not too worried about? Why not?
2. How important to you is the amount of time that it takes your child to feel better?
    a. Is there a point in time when you're not concerned versus a point in time when you become concerned?

For peer review only

For peer review only - http://bmjopen.bmj.com/site/about/guidelines.xhtml

Supplementary File 2

3. How concerned would you be if your child got sick again with the same illness, after feeling better?

- Sometimes when children are sick, they are not themselves. Respiratory infections can mean some nasty symptoms for kids, like coughs, fevers, and sore throats. What symptoms do you find most concerning when your child is sick? Tell us in the comments below!

- Parents, how do your dinosaurs get well soon?! Tell us what your kids are like when they're sick in the comments below!

- Respiratory infections can bring anyone down for a while. How do your kids cope? #BringBackFridayFunDay

Week 5: February 29 – March 4, 2016

Theme: **Financial Burden**

Sometimes when children are sick, parents or caregivers need to pay for extra things.

1. How concerned are you about the costs that come up when your child is sick?
    a. Costs related to treatment, like medications, supplies, or doctor or nurse visits?
    b. Other costs that came up while your child was sick, like child care, parking, or lost income for missing work?

- Happy Monday, Moms and Dads! This week, we're talking about costs that might come up when your kids are sick. Tell us what has come up for you in the comments below!

- There's a lot to think about when your child is sick. How much do you think about extra costs? Maybe that looks like costs for treatment, or maybe it's something like lost income for missing work. Share your thoughts in the comments below!

- Those darn hidden costs! Tell us what gets to you in the comments below!

Calvin and Hobbes, by Bill Watterson

Week 6: March 7 – 11, 2016

Theme: **Daily Activities and Routines**

Sometimes when children are sick, we can't follow a regular schedule or do regular activities.

For peer review only - http://bmjopen.bmj.com/site/about/guidelines.xhtml

Supplementary File 2

1. Are there any regular activities that you'd be concerned about if your child couldn't do them when they're sick and their breathing is affected?
   a. How concerned would you be about your child:
      i. Not eating or drinking well?
      ii. Not getting as much sleep as they normally do?
      iii. Being away from school or daycare?
   b. How concerned would you be about:
      i. Having to take time off work?
      ii. Arranging child care for your sick child or other children?
2. Are there any other activities that you'd be concerned about disruptions to?

- Welcome to another week of the OUTCH Study! Thanks for checking us out. This week, we're wondering about how your regular routine is disrupted when your child is sick. What happens in your family that's out of the ordinary? Tell us below!

- Sound familiar? Are there activities that you and your little ones miss when they are sick?

Just a Little Sick, by Mercer Mayer

- Thanks for the great discussion this week! Sounds like sleep is at the top of a lot of parents' lists of routines that are disrupted when their child is sick - although "everything" would also probably cover it! What else happens in your family? Tell us in the comments below!

<u>Week 7: March 14 – 18, 2016</u>

Theme: **Complications**

It can sometimes be scary when our children have health complications because their breathing is affected

1. How concerned would you be if your child had mild complications like a cough or a rash?
   a. What was or would be most concerning about your child having mild complications?
2. How concerned would you be if your child had severe complications like needing to be connected to a ventilator to help them breathe?
   a. What was or would be most concerning about your child having severe complications when they were sick and their breathing was affected?

- Welcome back, Moms and Dads! Sometimes when children are sick, complications come up, which can be scary. Complications might be things like a cough or a rash, or could be more serious like needing to

For peer review only - http://bmjopen.bmj.com/site/about/guidelines.xhtml

Supplementary File 2

be connected to a ventilator. Have your children gone through this? Please share your experience below.

- This week, we're talking about complications. Here's one mom's story:

He had all the signs of a cold coming on. With the rest of his siblings having been sick over the last few weeks, it was no surprise. He was super cranky all day and by supper time, he wasn't eating or drinking much. He'd had colds before but he wasn't acting well and looked tired. It all happened so fast - a drive to the hospital and 5 hours later, he was hooked up to a machine to help him breathe. The doctor said he had RSV and would likely need to stay a few days to be monitored and recover. I can't forget the helplessness I felt, watching him fighting to breathe, needing to be connected to the ventilator.

What about you? Please share your story in the comments below.

- Hey Parents, has your child had any complications when they've been sick with a respiratory infection? Tell us about it in the comments below.

<u>Week 8: March 21 – 25, 2016</u>

Theme: **Closing**

1. Please tell us any other things that are important to you when your child is sick with an illness that affects their breathing that we have not talked about yet.

- Happy Monday, everyone, and welcome to the last week of the OUTCH Study! Until next Sunday, tell us what matters to you when your child is sick with a respiratory infection. Is it their symptoms? Needing to see a healthcare provider? Disruptions to normal routines? Share what hits home for you in the comments below!

- Hey Parents, help us make sure we're not missing anything! What is most important to you when your child has a respiratory infection? Anything that's on your mind – we want to know!

- Thanks, everyone, for taking part in the OUTCH Study! Your input means a lot to us as we continue to try to make child health research and care better!

For peer review only - http://bmjopen.bmj.com/site/about/guidelines.xhtml

Completed STROBE Checklist for the Quantitative Component

	Item No	Recommendation	Page #
**Title and abstract**	1	(*a*) Indicate the study's design with a commonly used term in the title or the abstract	1
		(*b*) Provide in the abstract an informative and balanced summary of what was done and what was found	2
**Introduction**			
Background/rationale	2	Explain the scientific background and rationale for the investigation being reported	4-5
Objectives	3	State specific objectives, including any prespecified hypotheses	5
**Methods**			
Study design	4	Present key elements of study design early in the paper	5
Setting	5	Describe the setting, locations, and relevant dates, including periods of recruitment, exposure, follow-up, and data collection	7
Participants	6	(*a*) Give the eligibility criteria, and the sources and methods of selection of participants	5-6
Variables	7	Clearly define all outcomes, exposures, predictors, potential confounders, and effect modifiers. Give diagnostic criteria, if applicable	7
Data sources/ measurement	8*	For each variable of interest, give sources of data and details of methods of assessment (measurement). Describe comparability of assessment methods if there is more than one group	5-7; Supplement 1
Bias	9	Describe any efforts to address potential sources of bias	n/a
Study size	10	Explain how the study size was arrived at	8
Quantitative variables	11	Explain how quantitative variables were handled in the analyses. If applicable, describe which groupings were chosen and why	8-9
Statistical methods	12	(*a*) Describe all statistical methods, including those used to control for confounding	8-9
		(*b*) Describe any methods used to examine subgroups and interactions	n/a
		(*c*) Explain how missing data were addressed	n/a

For peer review only - http://bmjopen.bmj.com/site/about/guidelines.xhtml

		(*d*) If applicable, describe analytical methods taking account of sampling strategy	n/a
		(*e*) Describe any sensitivity analyses	n/a
**Results**			
Participants	13*	(a) Report numbers of individuals at each stage of study—eg numbers potentially eligible, examined for eligibility, confirmed eligible, included in the study, completing follow-up, and analysed	9
		(b) Give reasons for non-participation at each stage	n/a
		(c) Consider use of a flow diagram	n/a
Descriptive data	14*	(a) Give characteristics of study participants (eg demographic, clinical, social) and information on exposures and potential confounders	10-11 (Table 1)
		(b) Indicate number of participants with missing data for each variable of interest	10-11 (Table 1)
Outcome data	15*	Report numbers of outcome events or summary measures	11-13 (Table 2, Table 3, Table 4)
Main results	16	(*a*) Give unadjusted estimates and, if applicable, confounder-adjusted estimates and their precision (eg, 95% confidence interval). Make clear which confounders were adjusted for and why they were included	n/a
		(*b*) Report category boundaries when continuous variables were categorized	n/a
		(*c*) If relevant, consider translating estimates of relative risk into absolute risk for a meaningful time period	n/a
Other analyses	17	Report other analyses done—eg analyses of subgroups and interactions, and sensitivity analyses	n/a
**Discussion**			
Key results	18	Summarise key results with reference to study objectives	15-18
Limitations	19	Discuss limitations of the study, taking into account sources of potential bias or imprecision. Discuss both direction and magnitude of any potential bias	18
Interpretation	20	Give a cautious overall interpretation of results considering objectives, limitations, multiplicity of analyses, results from similar studies, and other relevant evidence	18

For peer review only - http://bmjopen.bmj.com/site/about/guidelines.xhtml

Generalisability	21	Discuss the generalisability (external validity) of the study results	18
**Other information**			
Funding	22	Give the source of funding and the role of the funders for the present study and, if applicable, for the original study on which the present article is based	19

For peer review only

For peer review only - http://bmjopen.bmj.com/site/about/guidelines.xhtml

BMJ Open

Which outcomes are important to patients and families who have experienced pediatric acute respiratory illness? Findings from a mixed methods sequential exploratory study

Journal:	*BMJ Open*
Manuscript ID	bmjopen-2017-018199.R2
Article Type:	Research
Date Submitted by the Author:	08-Nov-2017
Complete List of Authors:	Dyson, Michele; University of Alberta, Department of Pediatrics<br>Shave, Kassi; University of Alberta, Department of Pediatrics<br>Gates, Allison; University of Alberta, Department of Pediatrics<br>Fernandes, Ricardo; Hospital de Santa Maria, Departamento da Criança e da Família; Faculdade de Medicina de Lisboa, Instituto de Medicina Molecular, Laboratorio de Farmacologia Clinica e Terapeutica<br>Scott, Shannon; University of Alberta, Nursing<br>Hartling, Lisa; University of Alberta, Pediatrics
**Primary Subject Heading**:	Paediatrics
Secondary Subject Heading:	Patient-centred medicine, Respiratory medicine, Infectious diseases
Keywords:	Community child health < PAEDIATRICS, Paediatric infectious disease & immunisation < PAEDIATRICS, RESPIRATORY MEDICINE (see Thoracic Medicine), Paediatric thoracic medicine < THORACIC MEDICINE, Respiratory infections < THORACIC MEDICINE

SCHOLARONE™
Manuscripts

For peer review only

Which outcomes are important to patients and families who have experienced pediatric acute respiratory illness? Findings from a mixed methods sequential exploratory study

Michele P Dyson, Kassi Shave, Allison Gates, Ricardo M Fernandes, Shannon D Scott, Lisa Hartling

Michele P Dyson, Assistant Professor, Department of Pediatrics, University of Alberta

Kassi Shave, Former Research Assistant, Alberta Research Centre for Health Evidence (ARCHE), Department of Pediatrics, University of Alberta

Allison Gates, Research Associate, Alberta Research Centre for Health Evidence (ARCHE), Department of Pediatrics, University of Alberta

Ricardo M Fernandes, Pediatrician, Department of Pediatrics, Hospital de Santa Maria, Lisbon

Shannon D Scott, Professor, Faculty of Nursing, University of Alberta

Lisa Hartling, Professor, Department of Pediatrics, University of Alberta

Correspondence to: Dr. Michele P Dyson, 4-474 Edmonton Clinic Health Academy, University of Alberta, 11405 – 87 Avenue, Edmonton, Alberta, Canada T6G 1C9, E-mail: mdyson@ualberta.ca, Phone: 1.780.492.1241

For peer review only

For peer review only - http://bmjopen.bmj.com/site/about/guidelines.xhtml

ABSTRACT

Objectives. To identify the outcome priorities of parents of children who had experienced an acute respiratory infection (ARI).

Design. This was a two-phase, mixed methods study with a sequential exploratory design. We used a cross-sectional quantitative web-based survey to elicit parents' priorities for pediatric ARI. We then used a discussion moderated via Facebook to elucidate richer descriptions of parents' priorities.

Setting. Survey and discussion data were collected via the Internet.

Participants. 110 parents (90% female, median age 35 years, 92.7% urban dwelling, 94.5% with a post-secondary education) with a child who had experienced an ARI responded to the survey. Four parents participated in the Facebook discussion.

Primary and secondary outcome measures. The primary outcome was parents' rankings of outcomes related to pediatric ARI. The secondary outcomes were the alignment of parent-reported important outcomes with those commonly reported in Cochrane systematic reviews (SRs).

Results. Commonly reported ARIs included croup (44.5%), wheezing (43.6%), and influenza (38.2%). Parents ranked major complications, illness symptoms, and length of stay as the most important outcome categories. With respect to specific outcomes, severe complications, major side effects, doctor's assessment, relapse, oxygen supplementation, and results from lab measures were reported as most important (75th-99th percentile). Taking time off work, mild complications, interference with daily activities, treatment costs, absenteeism, follow-up visits and other costs were deemed minimally important (<25th percentile). In 35 Cochrane SRs, 29 unique outcomes were reported. Although participants' priorities sometimes aligned with outcomes frequently reported in the literature, this was not always true. Additional priorities from the survey (n=50) and Facebook discussions (n=4) included healthcare access, interacting with healthcare providers, education, impact on daily activities, and child wellbeing.

Conclusions. In the context of pediatric ARI, parents' priorities did not always align with commonly researched outcomes. Appealing and efficient strategies to engage patients and parents in research should be developed.

Key Words: social media, child health, patient-centered outcomes research, stakeholder engagement

For peer review only

STRENGTHS AND LIMITATIONS OF THIS STUDY

- The identification of patient-important outcomes is a necessary precursor to the conduct of research that is relevant to themselves and their families, though engaging patients in research is challenging.
- We used web-based tools and social media platforms to recruit and engage patients and identify patient-important outcomes for pediatric acute respiratory infections.
- Though nearly three-quarters of online adults use social media, engaging in health research online may appeal only to certain sub-populations, so the findings may not be generalizable.

For peer review only

For peer review only - http://bmjopen.bmj.com/site/about/guidelines.xhtml

INTRODUCTION

The determination of outcomes that matter to patients is foundational to the conduct of research that is relevant to them and their families. With an increasing emphasis on patient-centeredness in clinical research, numerous organizations and strategies have been established with this as their mandate (e.g., Canada's Strategy for Patient-Oriented Research [1], the Patient-Centered Outcomes Research Institute in the United States [2], and INVOLVE in the United Kingdom [3]). Involving patients in the research process will ensure that funded investigations utilize questions, outcomes, and interventions that are aligned with their needs and priorities [4-6].

There are many complexities involved in selecting outcomes, and little published guidance for investigators exists [7]. There is significant heterogeneity in the outcomes measured and reported in studies of specific diseases, which may in part occur due to uncertainty around which outcomes are patient-important [7]. The development of core outcome sets, in which a minimum group of agreed-upon outcomes is measured and reported on across clinical research in a specific condition, has been proposed as a solution to these issues [8]. Sinha et al. [7] identified 13 groups formed to develop core outcome sets for pediatric clinical trials, including the Core Outcome Measures in Effectiveness Trials (COMET) and Outcome Measures in Rheumatology (OMERACT). The COMET initiative was launched in 2010 to bring together individuals interested in developing core outcome sets, and to collate outcome sets and relevant resources [8]. Established in 1992, OMERACT is a consensus initiative that has developed a number of widely utilized core outcome sets for rheumatologic conditions, with patients actively involved in the process since 2002 [9, 10].

One criticism of commonly used methods to develop core outcome sets is that they do not include a systematic survey of stakeholders [9]. Social media represents a medium where patients and their caregivers increasingly interact online [11, 12], providing an opportune channel for engagement in the development of core outcomes. Nearly three quarters (74%) of online adults use social media, with Facebook continuing to be the most popular social media site, and multi-platform use increasing in prevalence [13, 14]. Despite the global pervasiveness of social media, its use for engaging patients and/or caregivers in the outcome selection process has not extensively been explored.

We conducted a two-phase, sequential exploratory mixed methods study using social media to recruit and engage the parents of children with an acute respiratory infection (ARI) to elucidate patient-

For peer review only - http://bmjopen.bmj.com/site/about/guidelines.xhtml

important outcomes. Acute respiratory infections are common among children, represent a significant source of morbidity, and are one of the leading causes of illness, emergency department visits, and hospitalization [15, 16]. There is a scarcity of research on patient perspectives in this area [7]. Using social media, we aimed to: (a) recruit and survey parents to identify their priorities for ARI outcomes as compared to those commonly reported in the literature, and (b) engage parents in discussions to elucidate the rationale for their priorities, as identified in the quantitative survey.

METHODS

This mixed methods study utilized an explanatory sequential design [17], and involved two phases: 1) a quantitative survey to determine parent priorities for ARI outcomes; 2) a qualitative follow-up in which we sought elaboration on parents' priorities. A process evaluation of our social media strategy is reported elsewhere [18].

Institutional Ethics Approval

Prior to beginning the study, we sought and received ethical approval for both the quantitative and qualitative components from the University of Alberta Research Ethics Board (# Pro00058629). Parents and/or caregivers were eligible to participate if they had a child aged 0-17 years who had experienced one or more episodes of acute asthma, bronchiolitis, croup, influenza, strep throat/tonsillitis, pneumonia, sinusitis, and/or wheezing. Participant consent was implied through overt action by completing the survey or publishing public responses online. Prior to participation, we provided interested potential participants with an information letter that described the study and explained consent via overt action. Participants were free to withdraw, end, or modify their participation in the study at any time without consequence, and we retained any data collected only with their permission.

Phase I: Quantitative Survey

Development and Pre-Testing

The first draft of the survey was informed by previous research on the outcomes that are important to clinicians and families of children with asthma [19] and by outcomes frequently reported in the literature. To determine the frequency of outcomes reported in the literature, we identified systematic reviews (SRs) published up to 2013 from the Cochrane Database of Systematic Reviews (n=35) and their included studies. We grouped frequently reported outcomes and those previously identified as important [7, 19, 20] into seven broad categories: disease activity; disease complications; adverse

For peer review only - http://bmjopen.bmj.com/site/about/guidelines.xhtml

For peer review only

effects of therapy; functional status; social and family outcomes, including quality of life; long-term effects of interventions; and resource utilization. We sent a draft of our survey to a group of 10 clinicians/researchers and 8 parents who reviewed it independently and provided feedback on its content and comprehensibility. We analysed the written feedback qualitatively. Based on the content analysis, our research team revised the original categories, finally deciding on ten categories of outcomes for the survey. These included outcomes that were identified as important by clinicians and parents that were missing from the draft. The ten categories on the final version of the survey included: major complications; symptoms; length of stay in the emergency department or hospital; needing to see a doctor; returns visits to a doctor or the hospital; reactions to medications; medical test results; maintenance of day-to-day activities; minor complications; and cost of illness. The survey is available as a supplementary file (Supplementary File 1).

We ensured survey accessibility across different operating systems, including touch screen (e.g., tablets, smartphones) or keyboard (e.g., desktop computers) technology. We wrote the materials at a sixth grade reading level and conducted pilot testing with 8 parents and 10 researchers/clinicians to ensure that the language and flow of questions were appropriate. We ascertained the reading level of our materials via the readability statistics provided in Microsoft Office Word's proofing options. With the readability statistics option turned on, Word returns the Flesch-Kincaid Grade Level of the document or the highlighted text following proofing for spelling and grammar.

Recruitment

We created an online and social media presence via a study website (www.outch-study.com), a Facebook page (OUTCH), and a Twitter account (@OUTCH_Study). We used snowball sampling [21] to recruit parents. First, we focused on identifying and engaging recruitment targets with the potential for a high yield of participants. We then expanded our scope through referrals and diffusion via social media. Tactica Interactive (http://tactica.ca), a digital media enterprise, was hired to broaden our sampling frame via a Facebook advertising strategy.

We collaborated with organizations interested in ARI and patient engagement to advertise our research via websites and other channels: The Alberta Centre for Child, Family & Community Research (ACCFCR, now known as PolicyWise for Children and Families; a provincial organization linking government, academia, and the community in a focus on evidence-informed policy and practice) [22], TRanslating

For peer review only - http://bmjopen.bmj.com/site/about/guidelines.xhtml

Emergency Knowledge for Kids (TREKK; a national network of researchers and clinicians invested in improving pediatric emergency care) [23]; the Cochrane Consumer Network (CCNet; an international network of healthcare consumers with an interest in evidence-based medicine) [24]; and the Stollery Family Centered Care Network (a local children's hospital-based network of patients and families that provide input into patient care) [25]. We also engaged an online (Facebook and Twitter) parenting community, Mommy Connections, that regularly promoted the study through their networks.

Data Collection

The quantitative survey was administered by Nooro Online Research (https://nooro.com/index.html) for 14 weeks from December 2013 to March 2014. Links to the survey were provided through the study website, Facebook, and Twitter accounts and were completed anonymously. The only identifying information was an optional e-mail address for entry into a prize draw.

The survey included a combination of open- and closed-ended questions to determine the relative importance of outcomes currently measured in trials and SRs of ARI in children. The outcome categories were presented and parents were asked to identify their top five priorities from the list. Then, individual outcomes were presented and parents were asked to indicate their importance using a digital sliding scale, conceptually similar to a visual analog scale. The sliding scale asked parents to rank the importance of each outcome by providing it with a score ranging from from 1 (not important [/concerning] at all) to 100 (extremely important [/concerning]). Parents were also asked to indicate additional items that were considered important to patients and their families, but may not have been addressed in the literature. The survey platform was also used to collect demographic data.

Phase II: Qualitative Follow-Up

In the second phase, we conducted an Internet-based, descriptive qualitative study [26] to interact with parents and elicit elaboration on their perceptions of the importance of ARI outcomes. This study occurred across an eight-week period from January to March 2016 during which parents were engaged in discussion through an open online focus group hosted on the study Facebook page.

Recruitment

To recruit parents, we utilized a snowball sampling technique [21]. We first asked potentially high yield sources of participants to promote our study, including local and national online parenting communities (n=16), children's hospitals and associated foundations (n=14), and patient groups (n=3) with access to a

For peer review only - http://bmjopen.bmj.com/site/about/guidelines.xhtml

large consumer audience. We also asked individuals and organizations within our existing networks to promote the study. Recruitment occurred throughout the study period, and was almost exclusively carried out through Facebook. Some organizations or individuals promoted the study on Twitter or a blog, but all links posted drove user traffic back to the study Facebook page.

One variable of interest in this study was the reach of social media as a recruitment strategy (reported elsewhere [18]); therefore, the sample size was an outcome, rather than a pre-defined condition. To accommodate this, we did not define the number of participants *a priori*, instead allowing the detail that emerged from our data collection to guide the extent of recruitment. However, we did aim to recruit a sample size as guided by the principles of data saturation, in which data would be collected until no new themes emerged [27].

Data Collection

Throughout the study period, each week had a discussion theme modelled after the structure of the survey, and posts were published daily covering varying aspects of this theme. Three different types of posts were published: promotional posts prior to the study launch; parent-friendly content about ARIs; and discussion questions where parents were encouraged to share their thoughts and experiences. The online focus group was moderated by two members of the study team (MPD, KS) and all posts by participants were followed up promptly with a response. An interview guide (Supplementary File 2) was developed to guide weekly topics and for reference during discussion moderation.

Data Analysis

Quantitative Analysis

Survey data were analyzed using SPSS (v. 22.0, IBM Corporation, Armonk, NY) and described using descriptive statistics (mean±SD; rank order). To determine the rank order of the outcomes most important to parents, we allocated each outcome 10 points when it was chosen as a top concern, and 8, 6, 4, and 2 points if it was chosen as the second, third, fourth, or fifth most important concern, respectively. After tallying the points for each of the 10 outcomes, we ordered these from largest to smallest to develop the ranked priority list.

To compare the published literature to the patient-important outcomes identified by parents, we collated a list of all of the outcomes, and calculated the number of SRS in which each outcome had been

For peer review only - http://bmjopen.bmj.com/site/about/guidelines.xhtml

reported. We then grouped all of the outcomes into percentile ranges based on the number of SRs in which each was reported ('frequently reported': 75th-99th percentile; 'moderately reported': 25th-74.9th percentile; and 'infrequently reported': <25th percentile). Similarly, we calculated the mean score given to each outcome by parents on the digital sliding scale (i.e., scores from 1 to 100), and grouped the outcomes into percentile ranges ('most important': 75th-99th percentile; 'moderately important': 25th-74.9th percentile; and 'least important': <25th percentile). All comparisons were strictly descriptive. Parents who had healthcare insurance were self-identified in the survey and excluded from all analyses related to healthcare costs as these may not hold relevance.

Qualitative Analysis

Content posted by participants during the focus groups was extracted verbatim to form transcripts. These, along with the open-ended responses to the survey, were imported into NVivo 10 (QSR International, Melbourne, Australia) data management software. Data were analyzed inductively for themes. Two investigators (MPD, KS) participated in coding, following a three stage process: 1) reading through the data, making notes on themes and significance that were then compiled into a preliminary version of the codebook; 2) rereading the data and coding using the concepts identified in the first phase; and 3) refining and applying the codes to the text upon a third review [28]. Coders met to discuss progress and reach consensus on differing interpretations. Data collection and analysis occurred concurrently, following an iterative process to monitor progress and allow for follow up on ideas as they emerged.

RESULTS

Demographic Characteristics

A total of 110 people responded to the survey (Table 1). The survey website received 5,207 visits, a view rate (i.e., the ratio of unique survey visitors/unique site visitors) of 3.9% (205/5,207), and a completion rate (i.e., the ratio of unique visitors who completed the survey/users who agreed to participate) of 53.7% (110/205). A detailed account of the traffic to the survey and usability are available in our published process evaluation [18].

Ninety percent (n=99) of respondents were female. The median age was 35 years, and 88% (n=97) of respondents had received a college/university or post-graduate education. Most survey respondents were married (n=98; 89%), urban-dwelling (n=102; 93%), and resided in Canada (n=77; 70%). More than

For peer review only - http://bmjopen.bmj.com/site/about/guidelines.xhtml

half of respondents reported an annual household income of >$90,000 USD (n= 69; 63%). Respondents were predominantly parents (n=106; 96%), and had a median of two children in the home (range 0-4). The respondents' children most commonly experienced croup (n=49; 45%), wheezing (n=48; 44%), and influenza (n=42; 38%). The most concerning ARIs were croup (n=20; 26%), pneumonia (n=16; 21%), and asthma (n=15; 19%). The median year that the ARI occurred was 2012 (range 1994-2013), when the child was 1-year-old (range <1 month-10 years old). Most children did not have a chronic illness (n=90; 82%) and did not experience a hospital admission due to this ARI (n=84; 76%).

Table 1. Survey participant demographics (N=110)

Characteristic	N (%)
Gender	Female: 99 (90.0)<br>Male: 11 (10.0)
Age (years) (median (range))	35 (18-67)
Highest level of education	Some high school: 1 (0.9)<br>High school graduate: 5 (4.5)<br>Some college/university: 7 (6.4)<br>College/university graduate: 50 (45.5)<br>Post-graduate education/degree: 47 (42.7)
Marital status	Single: 5 (4.5)<br>Married/Common-law: 98 (89.1)<br>Separated/divorced/widowed: 7 (6.4)
Annual household income (USD$)	<30,000: 5 (4.5)<br>30-49,999: 7 (6.4)<br>50-69,999: 15 (13.6)<br>70-89,999: 14 (12.7)<br>>90,000: 69 (62.7)
Country of residence	Australia: 2 (1.8)<br>Canada: 77 (70.0)<br>England: 8 (7.3)<br>India: 2 (1.8)<br>Portugal: 2 (1.8)<br>United States: 19 (17.3)
Type of community	Urban (≥10,000): 102 (92.7)<br>Rural (<10,000): 7(6.4)<br>Missing: 1 (0.9)
Number of children in home (median (range))	2 (0-4)
Relationship to child	Parent: 106 (96.4)<br>Step-parent: 0 (0)<br>Grandparent: 4 (3.6)<br>Other: 2 (1.8)
Type of ARI	Bronchiolitis: 29 (26.4)<br>Croup: 49 (44.5)

For peer review only - http://bmjopen.bmj.com/site/about/guidelines.xhtml

Characteristic	N (%)
	Strep throat/tonsillitis: 36 (32.7)<br>Wheezing: 48 (43.6)<br>Influenza: 42 (38.2)<br>Pneumonia: 24 (21.8)<br>Asthma: 29 (26.4)<br>Other: 23 (20.9)
Most concerning ARI	Bronchiolitis: 10 (13)<br>Croup: 20 (26)<br>Strep throat/tonsillitis: 3 (4)<br>Wheezing: 6 (8)<br>Influenza: 2 (3)<br>Pneumonia: 16 (21)<br>Asthma: 15 (19)<br>Other: 6 (8)
Year of ARI (median (range))	2012 (1994-2013)
Child age at time of ARI (median (range))	1 year (<1 month-10 years)
Hospital admissions due to ARI	Yes: 21 (19.1)<br>No: 84 (76.4)<br>Missing: 5 (4.5)
Chronic illness	Yes: 20 (18)<br>No: 90 (82)

ARI: Acute Respiratory Infection

Quantitative Outcome Rankings

The overall ranking of categorized outcomes is shown in Table 2. On average, parents ranked major complications from the child's illness (e.g., long-term disability), illness symptoms (e.g., coughing, fever, sore throat), and length of stay in the emergency department or hospital as the most important outcome categories. Of least importance were the costs of their child's illness (e.g., medicine or child care), minor complications from the child's illness (e.g., cough or rash), and maintenance of day-to-day activities. The overall ranking of individual outcomes revealed that parents were most concerned about severe complications (mean score on a scale from 1-100: 94.5), major side effects (86.7), and their doctor's assessment (83.9). Other costs (e.g., child care, parking, lost income) (31.3), scheduled follow-up visits (38.1), and school/daycare absenteeism (40.4) received the lowest mean scores. When parents were grouped according to ARI their child had experienced and their rankings of the importance of outcomes were compared, Spearman correlation coefficients revealed strong agreement, indicating that perceptions of importance were consistent across conditions (Table 3).

Table 2. Overall ranking of categorized items (N=110)

For peer review only - http://bmjopen.bmj.com/site/about/guidelines.xhtml

Rank Order[a]	Category
1	Major complications from child's illness (e.g., long-term disability)
2	Illness symptoms (e.g., coughing, fever, sore throat)
3	Length of stay in the emergency department or hospital
4	Child needing to see a doctor
5	Return visits to the doctor or hospital
6	Child's reaction to his or her medicine (e.g., side effects)
7	Child's medical test results
8	Maintenance of day-to-day activities
9	Minor complications from child's illness (e.g., cough or rash)
10	Costs of child's illness (e.g., medicine or child care)

[a]Ordered from most to least important to parents.

Table 3. Agreement between acute respiratory infection type and parent ranking of categorized outcomes (N=110)

Type of Acute Respiratory Infection	Spearman Correlation Coefficient*
Bronchiolitis	0.94
Croup	0.75
Strep Throat/Tonsillitis	0.66
Sinusitis	0.87
Wheezing	0.76
Influenza	0.66
Pneumonia	0.85
Asthma	0.67
Other	0.50

*0-0.2: poor/slight agreement; 0.2-0.4: fair agreement; 0.4-0.6: moderate agreement; 0.6-0.8: substantial agreement; 0.8-1: near perfect agreement

A comparison of the outcomes reported in the literature to those reported as important by parents is shown in Table 4. We extracted 221 outcomes from the 35 Cochrane SRs; the same outcomes were often reported in more than one SR. Out of the full list of reported outcomes, we isolated 29 individual outcomes each of which was reported in 1 to 26 of the SRs. Adverse events were the most frequently measured outcome (f=26; 11.8%) in our sample of Cochrane SRs, and similarly, severe complications (score: 94.5/100) and major side effects (86.7) were ranked as the most important (75th-99th percentile) to parents. Likewise, parents ranked returning to school/work and the cost of treatment as least important (40.4 and 44, respectively; <25th percentile), and these outcomes were infrequently measured in Cochrane SRs (f=2; <25th percentile).

For peer review only - http://bmjopen.bmj.com/site/about/guidelines.xhtml

There were many discrepancies between the outcomes measured in Cochrane SRs and the outcomes parents ranked as important. Relapse and the need for oxygen supplementation were ranked among the most important outcomes by parents (81.8 and 81.6, respectively; 75th-99th percentile), but were measured moderately frequently (f=8 and f=5, respectively; 25th-74.9^{th} percentile) in Cochrane SRs. Similarly, the results from lab measures were ranked highly by parents (81.4; 75th-99th percentile), but were infrequently reported in Cochrane SRs (f=4; 25th-74.9^{th} percentile). The need for medication was one of the most frequently reported outcomes in Cochrane reviews (f=13; 75th-99th percentile), but was ranked as moderately important by parents (55.1; 25th-74.9^{th} percentile). Admission rate was the second most frequently measured outcome in Cochrane SRs (f=19; 75th-99th percentile), yet was also ranked less favorably among parents (76.9; 25th-74.9^{th} percentile). While the doctor's assessment of how the child is doing was ranked as the third most important outcome (83.9; 75th-99th percentile) by parents, only clinical scores/symptom scores, one of three corresponding components of this outcome (clinical scores/symptom scores; patient improvement; observed response to treatment) was measured frequently (f=15; 75th-99th percentile) in Cochrane SRs. Patient improvement was measured moderately frequently (f=6; 25th-74.9^{th} percentile), and observed response to treatment was measured the least frequently of any outcome included in this study (f=1; <25th percentile).

Table 4. Parent ranking of individual outcomes compared to frequency of measurement in Cochrane systematic reviews

Outcomes reported in Cochrane systematic reviews (N=35)	Frequency of reporting	Parent ranking of importance of outcomes (N=110)	Mean Score ±SD (/100)
**75th-99th percentile**			
Adverse events (local and systemic)	26	Severe complications	94.5 ±14.5
Admission rate (hospital, ED, ICU)	19	Major side effect	86.7 ±18.9
Physical signs	18	Doctor's assessment	83.9 ±19.6
Clinical measures	17	Relapse	81.8 ±18.4
Clinical scores/symptom scores	15	Oxygen supplementation	81.6 ±21.6
Length of stay/time to discharge (hospital, ED, ICU)	15	Results from lab measures	81.4 ±19.4
Need for medication	13		
**25th-74.9^{th} percentile**			
Severity of symptoms	12	Length of hospital stay	78.6 ±22.1
Duration of symptoms	10	Trip to emergency department	77.6 ±23.9
Complications	9	Time to recovery	77.4 ±17.8
Rates of relapse	8	Hospital admission	76.9 ±23.7
Clinical treatment failure	7	Return healthcare visit	76.9 ±24.0
Patient improvement	6	Not eating/drinking well	65.9 ±22.8
Time to resolution of illness/time to recovery	6	Lack of sleep	63.2 ±20.0

For peer review only - http://bmjopen.bmj.com/site/about/guidelines.xhtml

Duration of oxygen supplementation	5	Length of stay in emergency department	62.4 ±26.1
Mortality	5	Minor side effect	55.1 ±24.8
Laboratory measures	4	Prescription for medication	55.1 ±25.6
Readmission	4	Appointment with GP/pediatrician	48.4 ±27.8
		Arranging child care	48.3 ±30.1
**<25th percentile**			
Clinical cure	3	Taking time off work	47.2 ±28.4
Compliance and tolerance	3	Mild complications	46.3 ±23.3
Quality of life/patients' well-being	3	Interference with daily activities	44.6 ±23.5
Return healthcare visits	3	Treatment costs	44.0 ±32.5
Return to school/work	2	School/daycare absenteeism	40.4 ±26.5
GP visits	2	Scheduled follow-up visits	38.1 ±24.8
Treatment cost	2	Other costs	31.3 ±31.3
Adverse events that necessitated discontinuation of treatment	1		
Sleep disturbance	1		
Parental perception of child's status	1		
Observed response to treatment	1		

Qualitative Synthesis

A total of 50 respondents provided qualitative responses on the survey. Four participants contributed to the discussion on the Facebook page. The thematic analysis revealed five main analytical themes relating to parents' priorities and concerns when their child had an ARI: 'accessing health care'; 'interacting with healthcare providers'; 'illness education'; 'impact of illness on daily activities'; and 'child wellbeing'.

Accessing Health Care

Though not expressed by all parents, having timely access to health care for their child was a primary concern for many. Being able to get the medical advice they needed, without encountering substantial waiting times was important. Parents expressed concern about recognizing the signs and symptoms of their child's disease process. Determining whether their child's symptoms were serious or minor was considered challenging. Parents lacked confidence in deciding when to seek medical attention, particularly when the child frequently experienced breathing problems. Overall, parents shared wanting to avoid making unnecessary health care visits, and expressed the importance of receiving helpful tips from health care providers for managing acute symptoms at home.

Interacting with Health Care Providers

For peer review only

For peer review only - http://bmjopen.bmj.com/site/about/guidelines.xhtml

When parents accessed health care for their child, they desired to be taken seriously by healthcare providers. Parents described concern about their child's healthcare provider being dismissive or uninterested in their chief complaints about their child's health. Strong communication with healthcare providers was widely valued by parents. Parents expressed wanting to feel heard, and to have a medical team that was both helpful and thorough in explaining their findings in a way that was direct and understandable.

Illness Education

Receiving education about their child's illness was widely regarded as important. Parents described requiring a complete understanding of what to expect, when to seek medical attention, and what the recovery time and process would be like. Information about the long-term impact of their child's health condition, and what effect recurrences or exacerbations might have, was regarded as of specific importance. When appropriate, parents regarded being presented with a variety of treatment options as critical. Parents expressed wanting to be involved in their child's recovery, and regarded education about their child's illness as essential to taking an active role.

Impact of Illness on Daily Activities

Parents described concern around interruption of their work schedules and sleeping routines. Being able to stay at home or in hospital with their child when they were unwell was important to parents, while not always possible when balancing financial and care responsibilities for other siblings. Disruption of sleep routines for parents and siblings was also described as concerning, particularly among parents of children with uncontrolled cough. Potential spread of their child's ARI between siblings and to the parents themselves was noted as a primary concern, and highly disruptive to daily activities.

Child Wellbeing

Parents shared their concern for their child's psychological wellbeing when they had an ARI. Concern about how their child was coping when they were unwell, particularly for those children with recurring or chronic ARI, was emphasized. Parents also expressed concern about how others treated their child when they were sick. One parent described concern over their child being treated like an 'invalid' when experiencing an acute asthma exacerbation.

DISCUSSION

For peer review only

For peer review only - http://bmjopen.bmj.com/site/about/guidelines.xhtml

Knowledge of patients' and their families' priorities is essential to guide the conduct of research that is relevant to themselves as well as to clinicians and policymakers [19]. Using social media, we engaged >100 parents over 14 weeks in a survey to elucidate the outcomes that they deemed most important with regard to pediatric ARIs. Parents' most important concerns included clinical outcomes like major complications, symptoms, and length of stay in the emergency department or hospital. Psychosocial outcomes, and the ability of the family to cope during a child's illness, were also important. Not surprisingly, parents who participated in the focus groups were also concerned about process measures, like wait times, communication with healthcare providers, and managing their child's care at home. Although parents did not explicitly make the link, research has indicated that certain care processes, e.g., patient-centeredness, may contribute to better health outcomes [29]. For example, family centered care is associated with improved clinical outcomes for children and greater satisfaction with care [30]. The approach is characterized by honest communication between families and healthcare providers; policies and procedures that are tailored to the needs of families and children; ensuring support for families and children; and empowering them to participate in care decisions [30].

The maintenance of day-to-day activities was of relatively low importance to parents, seemingly contradicting the highly ranked importance of major complications and long-term disability. A previous study of asthma outcomes found that parents were more concerned about the long-term compared to short-term beneficial and harmful effects of therapy [19]. Because we did not quantify the temporality of the outcomes on the survey or in the discussions, we can only presume that parents interpreted the maintenance of day-to-day activities as a short-term outcome resulting from relatively minor illness. Although major complications can result in long-term or permanent changes to daily routines, they could also be potentially life threatening. When presented with the possibility of serious complications that could limit long-term quality of life, the maintenance of one's daily routine may seem relatively unimportant.

Health, though difficult to define, encompasses not only an individual's physical condition, but also their emotional, and psychological wellbeing [31]. Our survey of 35 Cochrane SRs revealed that a diverse array of health outcomes are being measured and reported, many of which are not aligned with those that are important to parents. These findings reinforce the growing recognition that insufficient consideration is being paid to the selection of outcomes within clinical trials [32]. Specifically, the need for core outcome sets [33], and especially ones that incorporate patient-reported and patient-centered

For peer review only - http://bmjopen.bmj.com/site/about/guidelines.xhtml

outcomes [34] has garnered increased attention in recent years. More consistent reporting of outcomes for pediatric ARI will be necessary to facilitate evidence synthesis [32, 34], to enhance trustworthiness by reducing the risk of reporting bias [35], and to reduce research waste [36].

Good practice in clinical trials includes selecting a primary outcome that measures a clinically relevant and important treatment benefit [37]. Likewise, we found that the bulk of the research in child health focused on biological outcomes, with relatively little attention being paid the psychosocial impact of illness [20, 38, 39]. Still, there remains room in research for the measurement of outcomes important to patients and their families. Standards for Research in (StaR) Child Health, founded in 2009, brings together clinical and methodological experts to develop and promote the uptake of evidence-based guidance for child health research [40]. They assert that trialists should measure the effects of interventions more comprehensively; by measuring long-term outcomes and those that are relevant to decision-makers and families, the findings of trials will be of greater value [20].

Moving toward greater inclusion of patient-important outcomes in pediatric health research is challenged by the fact that children and their parents can be difficult to reach and engage. Given the pervasiveness of social media use via multiple platforms by patients and their caregivers [41], we postulated that this would provide an opportune medium to learn parents' perspectives. Though we experienced relative success in recruiting parents to complete the survey, qualitative engagement via the Facebook discussion group was more arduous. Moreover, despite moderate success in engaging parents, we did not gather any information from children themselves. Children have the right to participate in matters that affect their own lives [42], and can provide unique perspectives that cannot be elicited from their caregivers. Nevertheless, children also require protection, and the extent to which minors can understand and express their own healthcare needs remains controversial [42].

The challenges that we experienced are not unique. A review of studies that addressed the process of outcome selection identified only three studies that involved parents and none that involved children in the identification of pediatric patient-important outcomes [7]. In deciding which outcomes should be measured in pediatric ARI, it will be essential that stakeholders with varied perspectives, including parents, children, researchers, clinicians, and decision-makers convene and reach agreement on research priorities [32]. Suggested approaches like the Delphi technique and nominal group technique provide a means for stakeholders to reach unanimity on important outcomes in child health research

For peer review only - http://bmjopen.bmj.com/site/about/guidelines.xhtml

[7,32]. These are, however, time consuming, resource-intensive and are highly burdensome to participants, which may limit recruitment and engagement. Further guidance is required on consensus methods that are efficient and appealing to patients, families and other stakeholders. Methods that are understandable to children will need to be developed if researchers are to uphold the rights of minors to be involved in their own healthcare [42]. Reconciling children's and parents' perspectives, and the extent to which minors should be involved in the consensus process, requires further study.

Limitations

Our sample of parents and guardians were highly educated, many of whom had family incomes well above the national median, were mostly urban dwelling, and mainly Canadian, limiting the generalizability of the findings. For example, participants with lower incomes, or those residing in countries without publicly funded health care may have placed more importance on the cost of illness. As we did not provide any details to parents as to the temporality of the outcomes on our survey and in our discussions, we were not able to determine whether short- or long-term complications were more important to parents.

We had great difficulty engaging parents in the qualitative discussion and only elucidated responses from four participants. This seriously limited our ability to make informed inferences with regard to parents' quantitative ranking of the outcomes, leaving these mainly open to interpretation. For this reason, our understanding of the reasoning behind parents' ranking of the outcomes, and the content of the emergent qualitative themes are preliminary. Further work is required to develop a more comprehensive understanding of why some ARI-related outcomes are more important to parents than others.

CONCLUSIONS

The conduct and reporting of research of little relevance to the primary stakeholders represents a significant source of research waste, and appears prevalent in the context of pediatric ARI. The development of core outcome sets that include patient-important outcomes will facilitate evidence synthesis and reduce reporting bias, supporting the utility and trustworthiness of research findings. Future investigations are required to elucidate ways to make engagement in research more efficient and appealing to patients and their families.

For peer review only - http://bmjopen.bmj.com/site/about/guidelines.xhtml

ACKNOWLEDGEMENTS

The authors would like to thank Sarah Oberik-Olivieri for her graphic design work for this study.

COMPETING INTERESTS

All authors have completed the ICMJE uniform disclosure form at www.icmje.org/coi_disclosure.pdf and declare: no support from any organisations for the submitted work; no financial relationships with any organisations that might have an interest in the submitted work in the previous three years; no other relationships or activities that could appear to have influenced the submitted work.

FUNDING

This work was supported by KT Canada, grant number CIHR 87776 (SG-1) and Alberta Innovates Health Solutions, grant number 201400561. LH is supported in part by a Canadian Institutes of Health Research New Investigator Award. SS is a Canada Research Chair (Tier II) for Knowledge Translation in Child Health and is also supported by an Alberta Innovates Health Solutions Population Health Investigator Award. The funders had no role in study design, data collection and analysis, decision to publish, or preparation of the manuscript.

AUTHOR CONTRIBUTIONS

MPD and LH designed the study. MPD oversaw all aspects of the study's implementation. MPD and KS collected, analysed and interpreted the data with input from AG, LH, RMF, and SDS. All authors had full access to the data, and can take responsibility for the integrity of the data and the accuracy of the data analysis. KS and AG drafted the manuscript. MPD, LH, RMF and SDS reviewed the manuscript critically for intellectual content. All authors approved the version of the manuscript that was submitted to the journal. MPD affirms that the manuscript is an honest, accurate, and transparent account of the study being reported; that no important aspects of the study have been omitted; and that any discrepancies from the study as planned have been explained.

DATA SHARING STATEMENT

Anonymized data are available from the corresponding author upon reasonable request.

For peer review only

For peer review only - http://bmjopen.bmj.com/site/about/guidelines.xhtml

REFERENCES

1. Canadian Institutes of Health Research [Internet]. Strategy for Patient-Oriented Research. 2016 [cited 2017 May 1]. Available from: http://www.cihr-irsc.gc.ca/e/41204.html.
2. Patient-Centered Outcomes Research Institute [Internet]. 2016 [cited 2017 May 1]. Available from: http://www.pcori.org/.
3. National Institute for Health Research [Internet]. INVOLVE. 2015 [cited 2017 May 1]. Available from: www.invo.org.uk.
4. Frank L, Basch E, Selby JV. Patient-Centered Outcomes Research Institute. The PCORI perspective on patient-centered outcomes research. *JAMA* 2014;312(15):1513-4.
5. National Institute for Health Research [Internet]. INVOLVE. Exploring the impact of public involvement on the quality of research: examples. 2013 [cited 2017 May 1]. Available from: http://www.invo.org.uk/wp-content/uploads/2013/08/invoNETexamples2013.pdf.
6. Methodology Committee of the Patient-Centered Outcomes Research Institute (PCORI). Methodological standards and patient-centeredness in comparative effectiveness research: the PCORI perspective. *JAMA* 2012;307(15):1636-40.
7. Sinha I, Jones L, Smyth RL, et al. A systematic review of studies that aim to determine which outcomes to measure in clinical trials in children. *PLoS Med* 2008;5(4):e96.
8. COMET Initiative [Internet]. 2017 [cited 2017 May 1]. Available from: http://www.comet-initiative.org/.
9. Boers M, Kirwan JR, Wells G, et al. Developing core outcome measurement sets for clinical trials: OMERACT filter 2.0. *J Clin Epidemiol* 2014;67(7):745-53.
10. Boers M, Brooks P, Strand CV, et al. The OMERACT filter for Outcome Measures in Rheumatology. *J Rheumatol* 1998;25(2):198-9.
11. Fernandez-Luque L, Bau T. Health and social media: perfect storm of information. *Healthc Inform Res* 2015;21(2):67-73.
12. Fox S, Duggan M. Health Online 2013 [Internet]. 2013 [cited 2017 May 1]. Available from: http://www.pewinternet.org/files/old-media/Files/Reports/PIP_HealthOnline.pdf.
13. Duggan M, Ellison NB, Lampe C, Lenhart A, Madden M. Social Media Update 2014 [Internet]. 2015 [cited 2017 May 1]. Available from: http://www.pewinternet.org/2015/01/09/social-media-update-2014/.
14. Facebook [Internet]. Stats. 2017. [cited 2017 May 1]. Available from: http://newsroom.fb.com/company-info/.

For peer review only - http://bmjopen.bmj.com/site/about/guidelines.xhtml

15. Doan Q, Enarson P, Kissoon N, et al. Rapid viral diagnosis for acute febrile respiratory illness in children in the Emergency Department. *Cochrane Database Syst Rev* 2014;(9):CD006452.

16. Silka PA, Geiderman JM, Goldberg JB, et al. Demand on ED resources during periods of widespread influenza activity. *Am J Emerg Med* 2003;21(7):534-9.

17. Creswell JW, Plano Clark VL. Designing and conducting mixed methods research. Thousand Oaks, CA: Sage Publications; 2007.

18. Dyson MP, Shave K, Fernandes RM, et al. Outcomes in child health: Exploring the use of social media to engage parents in patient-centered outcomes research. *J Med Internet Res* 2017;19(3):e78.

19. Sinha IP, Gallagher R, Williamson PR, et al. Development of a core outcome set for clinical trials in childhood asthma: a survey of clinicians, parents, and young people. *Trials* 2012;13:103.

20. Sinha IP, Altman DG, Beresford MW, et al. Standard 5: selection, measurement, and reporting of outcomes in clinical trials in children. *Pediatrics* 2012;129 Suppl 3:S146-52.

21. Miles MB, Huberman AM. Qualitative data analysis: An expanded sourcebook. Thousand Oaks, CA: Sage Publications;1994.

22. PolicyWise for Children and Families [Internet]. About Us. 2017 [cited 2017 May 1]. Available from: https://policywise.com/about-us/.

23. TRanslating Emergency Knowledge for Kids (TREKK) [Internet]. About Us. 2017 [cited 2017 May 1]. Available from: http://trekk.ca/about.

24. Cochrane. Cochrane Consumer Network. 2017 [cited 2017 May 1]. Available from: http://consumers.cochrane.org/.

25. Alberta Health Services [Internet]. Family Centred Care (FCC). Stollery Children's Hospital. 2017 [cited 2017 May 1]. Available from: http://www.albertahealthservices.ca/services/page10093.aspx.

26. Sandelowski M. What's in a name? Qualitative description revisited. *Res Nurs Health* 2010;33(1):77-84.

27. Guest G, Bunce A, Johnson L. How many interviews are enough? An experiment with data saturation and variability. *Field Methods* 2006;18(1):59-82.

28. Guest G, MacQueen KM, Namey EE. Applied thematic analysis. Thousand Oaks, CA: Sage Publications; 2012.

29. Rathert C, Wyrwich MD, Austin Boren S. Patient-centered care and outcomes. A systematic review of the literature. *Med Care Res Rev* 2013;70:351-79.

For peer review only - http://bmjopen.bmj.com/site/about/guidelines.xhtml

30. Committee on Hospital Care; Institute for Patient- and Family-Centered Care. Patient- and family-centered care and the pediatrician's role. *Pediatrics* 2012;129:394-404.

31. Huber M, Knottnerus JA, Green L, et al. How should we define health? *BMJ* 2011;343:d4163.

32. Williamson PR, Altman DG, Blazeby JM, et al. Developing core outcome sets for clinical trials: issues to consider. *Trials* 2012;13:132.

33. Gargon E, Williamson PR, Altman DG, et al. The COMET initiative database: progress and activities update (2014). *Trials* 2015;16:515.

34. Macefield RC, Jacobs M, Korfage IJ, et al. Developing core outcomes sets: methods for identifying and including patient-reported outcomes (PROs). *Trials* 2014;15:49.

35. Dwan K, Gamble C, Williamson PR, et al. Systematic review of the empirical evidence of study publication bias and outcome reporting bias - an updated review. *PLoS One* 2013;8(7):e66844.

36. Chalmers I, Bracken MB, Cjulbegovic B, et al. How to increase value and reduce waste when research priorities are set. *Lancet* 2014;383(9912):156-65.

37. International Conference on Harmonisation of Technical Requirements for Registration of Pharmaceuticals for Human Use (ICH). Guidance for Industry. E9 Statistical Principles for Clinical Trials. Rockville, MD: US Department of Health and Human Services; Food and Drug Administration; Center for Drug Evaluation and Research; Center for Biologics Evaluation and Research; 1998.

38. Clarke SA, Eiser C. The measurement of health-related quality of life (QOL) in paediatric clinical trials: a systematic review. *Health Qual Life Outcomes* 2004;2:66.

39. Sinha IP, Williamson PR, Smyth RL. Outcomes in clinical trials of inhaled corticosteroids for children with asthma are narrowly focussed on short term disease activity. *PLoS One* 2009;4(7):e6276.

40. Van't Hoff W, Offringa M, Star Child Health group. StaR Child Health: developing evidence-based guidance for the design, conduct and reporting of paediatric trials. *Arch Dis Child* 2015;100:189-92.

41. Hamm MP, Chisholm A, Shulhan J, et al. Social media use among patients and caregivers: a scoping review. *BMJ Open* 2013;3(5):e002819.

42. Söderbäck M, Coyne I, Harder M. The importance of including both a child perspective and the child's perspective within health care settings to provide truly child-centred care. *J Child Health Care* 2011;15(2):99-106.

For peer review only

For peer review only - http://bmjopen.bmj.com/site/about/guidelines.xhtml

Supplementary File 1

1. **We are interested in learning which things you would be most concerned about if your child was sick with an illness that affected their breathing. By moving each item from the left side of the screen to the right side of the screen, please choose the top five things that would be most concerning to you. In your list, put the thing that would be most concerning to you at the top, followed by the next most concerning thing, and so on.** *(randomize order)*

__ If your child needed to see a doctor
__ How long your child needed to stay in the emergency department or hospital
__ If your child needed to return to the doctor or hospital after being sent home
__ Your child's test results that tell the doctor how he or she is doing
__ Your child's reaction to his or her medicine (for example, if it caused side effects)
__ Your child's symptoms (for example, coughing, fever, sore throat)
__ The costs of your child's illness (for example, for medicine or child care)
__ How well you and your child could keep up the day-to-day activities of your normal life
__ That your child could develop minor complications from their illness, such as a cough or rash
__ That your child could have major complications from their illness, such as a long-term disability

---

We would like to further explore the things that matter to you, as a parent, when your child is sick with an illness that affects their breathing. For the questions below, use the sliding scale to show how concerned you would be in each of the situations described.

Not at all concerned---*Very concerned* __ *Can't answer*
(this would not be an important concern for you if your child was sick) (this would be a very important concern for you if your child was sick)

<u>**Getting Medical Help**</u>

Sometimes when children are sick and their breathing is affected, they need help from a doctor. How concerned would you be about the following: *(Image 1)*

1. That you had to take your child to your family doctor or pediatrician's office.
2. That you had to take your child to the emergency department.
3. How long you had to stay at the emergency department.
4. That your child had to stay in the hospital (overnight or longer).
5. How long your child had to stay in the hospital.
6. That your child needed a scheduled follow-up visit, either with your family doctor/pediatrician or at the hospital.
7. That you had to go back see a doctor after the first time because he or she was not getting better. This could be at a doctor's office, a clinic, or the hospital.

<u>**Tests and Treatment**</u>

Sometimes children need tests when they are sick. How important are the following things to you: *(sliding scale: Not at all important – Very important) (Image 4)*

For peer review only

Supplementary File 1

8. Assessment of things like how fast your child was breathing, how fast his or her heart was beating, or whether he or she had a fever.
9. Results from x-rays, blood tests, or other tests, showing how well your child's lungs were working or what might be causing their illness.
10. The doctor's assessment of how your child was doing.

Sometimes when children are sick, they need to take medicine or be treated in other ways. How concerned would you be if: *(Image 5)*

11. Your child needed to be given extra oxygen through a mask or through his or her nose.
12. Your child needed to take medicine to feel better.
13. Your child had a minor but common side effect from the treatment or medicine. Some common side effects are vomiting, rash, and diarrhea.
14. Your child had a major side effect from the treatment or medicine that was bad enough to make him or her stop taking it. Some such reactions are your child becoming very agitated or irritable, or having a serious allergic reaction.

__**Other Concerns**__

Sometimes when children are sick, they are not themselves. How concerned would you be about the following things if your child was sick? *(Image 6)*

15. How bad your child's symptoms were. Symptoms are signs of your child's sickness. Common symptoms are things like coughing, wheezing, fever, sore throat, headache, and struggling to breathe.
16. How long it took for your child to feel better.
17. If your child got sick again with the same illness, after feeling better.

Sometimes when children are sick, parents or caregivers need to pay for extra things. How concerned would you be about: *(Image 7)*

18. How much your child's treatment cost, including costs for things related to treating their illness like medications, supplies, or doctor or nurse visits.
19. Other costs that came up while your child was sick, for example, child care, parking, or lost income for missing work.

Sometimes when children are sick, parents or caregivers can't follow their regular schedule or do regular activities. How concerned would you be about: *(Image 8)*

20. Your child not eating or drinking well because they felt sick.
21. You or your child not getting as much sleep as you normally do.
22. Your child being away from school or daycare.
23. The illness affecting the activities your child, you, or your family would normally do on a day-to-day basis.
24. Having to take time off of work.
25. Arranging child care either for your child who was sick, or arranging child care for your other children so that you could look after your sick child or take him or her to appointments.

For peer review only

Supplementary File 1

It can sometimes be scary when our children are sick. *(Image 9)*

26. How concerned would you be if your child had mild complications such as a cough or a rash?
27. How concerned would you be if your child had severe complications? This could mean something like your child being connected to a ventilator to help his or her breathing.

---

28. **Please tell us any other things that are important to you when your child is sick with an illness that affects their breathing that have not been included in this list.**

Please tell us a little bit more about your family.

29. **What is your gender?**
    __ Male
    __ Female
    (__ NR)

30. **What is your relationship to the child(ren) in your family? Check all that apply.**
    __ Parent
    __ Step-parent
    __ Grandparent
    __ Other, specify: __________
    (__ NR)

31. **How many children live in your home?** ____ (__ NR)

32. **What illness or illnesses that affect breathing has your child or children ever had? Please check all that apply.**
    __ Bronchiolitis
    __ Croup
    __ Strep throat (pharyngitis) or tonsillitis
    __ Sinus infection (sinusitis)
    __ Wheezing
    __ Flu (influenza)
    __ Pneumonia
    __ Acute asthma
    __ Other, specify: ______________
    __ I don't remember the name of the illness
    (__ NR)

    33a. If you checked more than one illness above, which one did your child have **most recently**?
    (drop down menu – same list as above) (__ NR)

For peer review only

Supplementary File 1

*****The following three questions will appear to correspond to conditions above*****

33b**. How old was your child when they had this illness?** *(drop down menu - <1/1/…/10/11 months/1/2/…18 years/can't remember)* (__ NR)

33b. When did this illness occur? MM/YYYY (__ NR)

33c. **Was your child admitted to the hospital, to stay overnight or longer?**
- __ Yes
- __ No
- (__ NR)

33d. **Does your child have any health concerns?**
- __ Yes, details: ________________
- __ No
- (__ NR)

33. **What year were you born in?** 19____ (__ NR)

34. **What is the highest level of schooling you have completed?**
- __ Grades 1-9
- __ Grades 10-11/Some high school
- __ High school graduate
- __ Some college/university
- __ College/university graduate
- __ Post-graduate education or degree
- (__ NR)

35. **What is your marital status?**
- __ Never married (single)
- __ Married/Common-law
- __ Separated, divorced, or widowed
- __ Other, specify: _________
- (__ NR)

36. **What is your household income (US dollars) per year?** (http://www.xe.com/currencyconverter/)
- __ Less than $30,000 per year
- __ $30,000-49,999
- __ $50,000-69,999
- __ $70,000-89,999
- __ Over $90,000
- (__ NR)

37. **What was your place of birth (town/city, country)?** _________ ________ (__ NR)

38. **Where do you live now (town/city, country)?** _______ ________ (__ NR)

For peer review only

Supplementary File 1

39. **How did you hear about this survey?**

__ Facebook
__ Twitter
__ Internet search (for example, Google)
__ Consumer or patient group (for example, Cochrane Consumer Network)
__ From a friend
__ From a health care professional (for example, a doctor or nurse)
__ Research network (for example, TREKK [Translating Emergency Knowledge for Kids])
__ Other, specify: ____________________
(__ NR)

Thank you for taking the time to fill out this survey!

If you would like to be entered into a draw for an iPad mini, a Kobo Touch, or a Nike+ FuelBand, click here to provide us with your email address. Your email address will not be linked to the responses you have provided.

For peer review only

Supplementary File 2

OUTCH Discussion Guide

Important Considerations

1. Is there agreement on the outcomes chosen, both among parents involved in the discussion, and between the discussion and the survey?
2. Are there areas (i.e., in current research) that have been overlooked?
3. What is the rationale for outcome selection?
4. What are considered to be clinically important or meaningful differences in the outcomes selected?

Week 1: February 1 – 5, 2016

Theme: **Getting Medical Help**

Sometimes when children are sick and their breathing is affected, they need help from a doctor.

1. Tell us about a time you needed to bring your child to a doctor because their breathing was affected.
   a. Did you have to go to see a doctor? Go to the emergency department?
   b. How did you feel?
   c. Did, or would, one have more impact on you than the other?
2. Tell us about what you find most concerning about getting medical help for your child when they are sick and their breathing is affected.
   a. Possible considerations (as prompts):
      i. how long you had to stay at the emergency department;
      ii. how long you had to stay in the hospital;
      iii. if you had to go for a scheduled follow-up visit to your family doctor or pediatrician;
      iv. if you had to go back to see a doctor after the first time because your child wasn't getting better.

- Thanks for checking out the OUTCH Study! We want to make sure that your child's health care is based on what's most important to you when their breathing is affected. This week, tell us what matters when your child needs to see the doctor. [attach graphic]

- Hey Parents! Have you had to take your child to the doctor or emergency department because they were having a hard time breathing? What was important to you?

- Excited to get this discussion going about what is important to you when your child is having a hard time breathing! Check out our page and share your experiences in the comments section.

Week 2: February 8 – 12, 2016

Theme: **Medical Tests**

Sometimes children need tests and treatment when they are sick.

For peer review only - http://bmjopen.bmj.com/site/about/guidelines.xhtml

Supplementary File 2

1. Tell us about a time your child needed tests because they were sick and their breathing was affected.
    a. What kind of tests did they need?
    b. At the time, how much did the results of things like x-rays or blood tests matter to you?
    c. How much did the doctor's assessment of your child's illness matter to you?
    d. Did, and why did, one type of assessment matter more to you than the other?

- Welcome to another week of the OUTCH Study! Help us understand your story. Tell us in the comments below about what matters to you when your child needs medical tests because they are having a hard time breathing. [attach graphic]

- If your child has needed tests like x-rays or blood tests when they're sick, what has mattered to you? Tell us in the comments below!

- Get to Know OUTCH!
We're asking you to share your stories with us, so we thought we'd tell you a little more about our team!

What does OUTCH stand for?
OUTCH stands for Outcomes in Child Health.

Who are we?
We're a group of researchers from the Department of Pediatrics at the University of Alberta, and we're the ones behind the scenes at the OUTCH Study.

What is the OUTCH Study all about?
We're interested in learning about outcomes that matter to you when your children's breathing is affected. This could mean that they have, or had, an illness like bronchiolitis, croup, strep throat or tonsillitis, a sinus infection, wheezing, the flu, pneumonia, or acute asthma. We want to talk to parents like you to make sure that our research asks the right questions, so we can improve healthcare for children and their families.

How can you help?
Tell us what matters to you in the comments section of our Facebook posts – we'd love to hear from you!

<u>Week 3: February 16 – 19, 2016 (*Family Day on February 15)</u>

Theme: **Medical Treatment**

Sometimes when children are sick, they need to take medicine or be treated in other ways.

For peer review only - http://bmjopen.bmj.com/site/about/guidelines.xhtml

Supplementary File 2

1. Tell us about a time when your child was sick and they needed treatment because their breathing was affected.
    a. What kind of treatment did they need?
        i. Were they given medicine? Oxygen?
2. How concerned were you about your child needing treatment?
    a. What was most concerning about your child needing treatment because their breathing was affected?
3. If your child has had more than one kind of treatment, have you found certain treatments more concerning than others?
4. How concerned would you be concerned about a mild side effect from the treatment or medicine, like vomiting, a rash, or diarrhea?
5. How concerned would you be about a major side effect from the treatment or medicine (like being very agitated or having a serious allergic reaction), that was bad enough that your child had to stop taking it?

- Welcome back parents! Here in Alberta we're just coming off of our Family Day long weekend, but we hope you had a relaxing weekend wherever you are! This week, we're talking about medical treatment for your kids. What was your experience when your child was sick and needed treatment to help their breathing? What went well? What could have been better? What worried you? What was reassuring? Please tell us in the comments below!

- Medicine can help our kids a lot, but sometimes it comes along with annoying, or even scary, side effects. Has your child gone through this? Did it get bad enough to stop treatment, or were they okay to wait it out? Any lasting effects? A combination? Share your experience and how you felt in the comments below.

- What goes through your mind when your child is prescribed a treatment? Relief? Possible long-term effects? That it's the right one? A combination? Tell us your thoughts in the comments below!

<u>Week 4: February 22 – 26, 2016</u>

Theme: **Course of Illness**

Sometimes when children are sick, they are not themselves.

1. Tell us about symptoms you find the most concerning when your child is sick and their breathing is affected. *(Common symptoms are things like coughing, wheezing, fever, sore throat, headache, and struggling to breathe)* Why?
    a. What about things you're not too worried about? Why not?
2. How important to you is the amount of time that it takes your child to feel better?
    a. Is there a point in time when you're not concerned versus a point in time when you become concerned?

For peer review only - http://bmjopen.bmj.com/site/about/guidelines.xhtml

Supplementary File 2

3. How concerned would you be if your child got sick again with the same illness, after feeling better?

- Sometimes when children are sick, they are not themselves. Respiratory infections can mean some nasty symptoms for kids, like coughs, fevers, and sore throats. What symptoms do you find most concerning when your child is sick? Tell us in the comments below!

- Parents, how do your dinosaurs get well soon?! Tell us what your kids are like when they're sick in the comments below!

- Respiratory infections can bring anyone down for a while. How do your kids cope? #BringBackFridayFunDay

Week 5: February 29 – March 4, 2016

Theme: **Financial Burden**

Sometimes when children are sick, parents or caregivers need to pay for extra things.

1. How concerned are you about the costs that come up when your child is sick?
   a. Costs related to treatment, like medications, supplies, or doctor or nurse visits?
   b. Other costs that came up while your child was sick, like child care, parking, or lost income for missing work?

- Happy Monday, Moms and Dads! This week, we're talking about costs that might come up when your kids are sick. Tell us what has come up for you in the comments below!

- There's a lot to think about when your child is sick. How much do you think about extra costs? Maybe that looks like costs for treatment, or maybe it's something like lost income for missing work. Share your thoughts in the comments below!

- Those darn hidden costs! Tell us what gets to you in the comments below!

Calvin and Hobbes, by Bill Watterson

Week 6: March 7 – 11, 2016

Theme: **Daily Activities and Routines**

Sometimes when children are sick, we can't follow a regular schedule or do regular activities.

For peer review only - http://bmjopen.bmj.com/site/about/guidelines.xhtml

For peer review only

Supplementary File 2

1. Are there any regular activities that you'd be concerned about if your child couldn't do them when they're sick and their breathing is affected?
    a. How concerned would you be about your child:
        i. Not eating or drinking well?
        ii. Not getting as much sleep as they normally do?
        iii. Being away from school or daycare?
    b. How concerned would you be about:
        i. Having to take time off work?
        ii. Arranging child care for your sick child or other children?
2. Are there any other activities that you'd be concerned about disruptions to?

- Welcome to another week of the OUTCH Study! Thanks for checking us out. This week, we're wondering about how your regular routine is disrupted when your child is sick. What happens in your family that's out of the ordinary? Tell us below!

- Sound familiar? Are there activities that you and your little ones miss when they are sick?

Just a Little Sick, by Mercer Mayer

- Thanks for the great discussion this week! Sounds like sleep is at the top of a lot of parents' lists of routines that are disrupted when their child is sick - although "everything" would also probably cover it! What else happens in your family? Tell us in the comments below!

Week 7: March 14 – 18, 2016

Theme: **Complications**

It can sometimes be scary when our children have health complications because their breathing is affected

1. How concerned would you be if your child had mild complications like a cough or a rash?
    a. What was or would be most concerning about your child having mild complications?
2. How concerned would you be if your child had severe complications like needing to be connected to a ventilator to help them breathe?
    a. What was or would be most concerning about your child having severe complications when they were sick and their breathing was affected?

- Welcome back, Moms and Dads! Sometimes when children are sick, complications come up, which can be scary. Complications might be things like a cough or a rash, or could be more serious like needing to

For peer review only - http://bmjopen.bmj.com/site/about/guidelines.xhtml

Supplementary File 2

be connected to a ventilator. Have your children gone through this? Please share your experience below.

- This week, we're talking about complications. Here's one mom's story:

He had all the signs of a cold coming on. With the rest of his siblings having been sick over the last few weeks, it was no surprise. He was super cranky all day and by supper time, he wasn't eating or drinking much. He'd had colds before but he wasn't acting well and looked tired. It all happened so fast - a drive to the hospital and 5 hours later, he was hooked up to a machine to help him breathe. The doctor said he had RSV and would likely need to stay a few days to be monitored and recover. I can't forget the helplessness I felt, watching him fighting to breathe, needing to be connected to the ventilator.

What about you? Please share your story in the comments below.

- Hey Parents, has your child had any complications when they've been sick with a respiratory infection? Tell us about it in the comments below.

<u>Week 8: March 21 – 25, 2016</u>

Theme: **Closing**

1. Please tell us any other things that are important to you when your child is sick with an illness that affects their breathing that we have not talked about yet.

- Happy Monday, everyone, and welcome to the last week of the OUTCH Study! Until next Sunday, tell us what matters to you when your child is sick with a respiratory infection. Is it their symptoms? Needing to see a healthcare provider? Disruptions to normal routines? Share what hits home for you in the comments below!

- Hey Parents, help us make sure we're not missing anything! What is most important to you when your child has a respiratory infection? Anything that's on your mind – we want to know!

- Thanks, everyone, for taking part in the OUTCH Study! Your input means a lot to us as we continue to try to make child health research and care better!

For peer review only - http://bmjopen.bmj.com/site/about/guidelines.xhtml

Completed STROBE Checklist for the Quantitative Component

	Item No	Recommendation	Page #
**Title and abstract**	1	(*a*) Indicate the study's design with a commonly used term in the title or the abstract	1
		(*b*) Provide in the abstract an informative and balanced summary of what was done and what was found	2
**Introduction**			
Background/rationale	2	Explain the scientific background and rationale for the investigation being reported	4-5
Objectives	3	State specific objectives, including any prespecified hypotheses	5
**Methods**			
Study design	4	Present key elements of study design early in the paper	5
Setting	5	Describe the setting, locations, and relevant dates, including periods of recruitment, exposure, follow-up, and data collection	7
Participants	6	(*a*) Give the eligibility criteria, and the sources and methods of selection of participants	5-6
Variables	7	Clearly define all outcomes, exposures, predictors, potential confounders, and effect modifiers. Give diagnostic criteria, if applicable	7
Data sources/ measurement	8*	For each variable of interest, give sources of data and details of methods of assessment (measurement). Describe comparability of assessment methods if there is more than one group	5-7; Supplement 1
Bias	9	Describe any efforts to address potential sources of bias	n/a
Study size	10	Explain how the study size was arrived at	8
Quantitative variables	11	Explain how quantitative variables were handled in the analyses. If applicable, describe which groupings were chosen and why	8-9
Statistical methods	12	(*a*) Describe all statistical methods, including those used to control for confounding	8-9
		(*b*) Describe any methods used to examine subgroups and interactions	n/a
		(*c*) Explain how missing data were addressed	n/a

For peer review only - http://bmjopen.bmj.com/site/about/guidelines.xhtml

		(*d*) If applicable, describe analytical methods taking account of sampling strategy	n/a
		(*e*) Describe any sensitivity analyses	n/a
**Results**			
Participants	13*	(a) Report numbers of individuals at each stage of study—eg numbers potentially eligible, examined for eligibility, confirmed eligible, included in the study, completing follow-up, and analysed	9
		(b) Give reasons for non-participation at each stage	n/a
		(c) Consider use of a flow diagram	n/a
Descriptive data	14*	(a) Give characteristics of study participants (eg demographic, clinical, social) and information on exposures and potential confounders	10-11 (Table 1)
		(b) Indicate number of participants with missing data for each variable of interest	10-11 (Table 1)
Outcome data	15*	Report numbers of outcome events or summary measures	11-13 (Table 2, Table 3, Table 4)
Main results	16	(*a*) Give unadjusted estimates and, if applicable, confounder-adjusted estimates and their precision (eg, 95% confidence interval). Make clear which confounders were adjusted for and why they were included	n/a
		(*b*) Report category boundaries when continuous variables were categorized	n/a
		(*c*) If relevant, consider translating estimates of relative risk into absolute risk for a meaningful time period	n/a
Other analyses	17	Report other analyses done—eg analyses of subgroups and interactions, and sensitivity analyses	n/a
**Discussion**			
Key results	18	Summarise key results with reference to study objectives	15-18
Limitations	19	Discuss limitations of the study, taking into account sources of potential bias or imprecision. Discuss both direction and magnitude of any potential bias	18
Interpretation	20	Give a cautious overall interpretation of results considering objectives, limitations, multiplicity of analyses, results from similar studies, and other relevant evidence	18

For peer review only - http://bmjopen.bmj.com/site/about/guidelines.xhtml

Generalisability	21	Discuss the generalisability (external validity) of the study results	18
**Other information**			
Funding	22	Give the source of funding and the role of the funders for the present study and, if applicable, for the original study on which the present article is based	19

For peer review only

For peer review only - http://bmjopen.bmj.com/site/about/guidelines.xhtml
